# High glucose environment inhibits cranial neural crest survival by activating excessive autophagy in the chick embryo

**DOI:** 10.1038/srep18321

**Published:** 2015-12-16

**Authors:** Xiao-Yu Wang, Shuai Li, Guang Wang, Zheng-Lai Ma, Manli Chuai, Liu Cao, Xuesong Yang

**Affiliations:** 1 Division of Histology & Embryology, Key Laboratory for Regenerative Medicine of the Ministry of Education, Medical College, Jinan University, Guangzhou 510632, China; 2 Division of Cell and Developmental Biology, University of Dundee, Dundee, DD1 5EH, UK; 3 Key Laboratory of Medical Cell Biology, China Medical University, Shengyang 110001, China; 4 Division of Pathophysiology, Medical College, Jinan University, Guangzhou 510632, China

## Abstract

High glucose levels induced by maternal diabetes could lead to defects in neural crest development during embryogenesis, but the cellular mechanism is still not understood. In this study, we observed a defect in chick cranial skeleton, especially parietal bone development in the presence of high glucose levels, which is derived from cranial neural crest cells (CNCC). In early chick embryo, we found that inducing high glucose levels could inhibit the development of CNCC, however, cell proliferation was not significantly involved. Nevertheless, apoptotic CNCC increased in the presence of high levels of glucose. In addition, the expression of apoptosis and autophagy relevant genes were elevated by high glucose treatment. Next, the application of beads soaked in either an autophagy stimulator (Tunicamycin) or inhibitor (Hydroxychloroquine) functionally proved that autophagy was involved in regulating the production of CNCC in the presence of high glucose levels. Our observations suggest that the ERK pathway, rather than the mTOR pathway, most likely participates in mediating the autophagy induced by high glucose. Taken together, our observations indicated that exposure to high levels of glucose could inhibit the survival of CNCC by affecting cell apoptosis, which might result from the dysregulation of the autophagic process.

Gestational diabetes is characterized by either high blood glucose levels or glucose intolerance during pregnancy, and approximately 80% of diabetic pregnancies fall into this category^1^. This condition is usually diagnosed at 24–28 weeks of gestation, after the important periods for organogenesis have already passed. Thus, the maternal high glucose concentration could have already adversely affected the early development of the fetus. It has been reported that maternal hyperglycemia can result in many abnormalities such as macrosomia and developmental retardation^2^. Elevated glucose concentrations also negatively affect cardiogenesis and neurogenesis. In the central nervous system, high glucose levels can lead to neural tube defects (NTDs), such as exencephaly, anencephaly and rachischisis^3^,^4^. In addition, up to 17% of neonates and fetuses from diabetic mothers suffer congenital heart diseases, including atrioventricular septal defect and tetralogy of Fallot^5^.

In recent years, scientists have noticed that some tissues and organs derived from the neural crest, such as the cranial ganglia and the outflow tract, were involved in the fetal anomalies induced by maternal hyperglycemia^6^,^7^,^8^, which suggests that hyperglycemia impairs neural crest development and could ultimately lead to malformation. The neural crest cells (NCCs) are derived from the neural plate border (NPB), which is a population of pluripotent cells that undergoes induction, maintenance, delamination, epithelial-mesenchymal transition, migration, and can contribute to almost every organ system in vertebrates^9^. The cranial neural crest cells (CNCC) contribute to many tissues and organs, including the craniofacial skeleton, the cerebral ganglion of the sensory nervous system, the enteric nervous system, the Schwann cells, and the aortic wall^10^,^11^. The abnormal development of the neural crest can result in congenital malformations, such as NTDs, atrioventricular septal defects, patent ductus arteriosus, and Waardenburg’s syndrome.

Fetuses from diabetic mothers show severe neural tube defects such as anencephaly and exencephaly, which indicates that the development of not only the neural system but also the cranial skeleton is impaired^12^. The most studied mechanism for this is the production of excess reactive oxygen species (ROS) when the embryo is exposed to a hyperglycemic environment. Cranial neural crest cells are more sensitive to ROS than trunk neural crest cells^13^. It has been reported that the expression of Pax3, which encodes an important transcription factor in neural crest cells, is inhibited due to the oxidative stress induced by maternal hyperglycemia^14^,^15^.

At the same time, high glucose levels can induce autophagy^16^. Autophagy is a protective process in cells that is intended to maintain homeostasis under normal conditions. During autophagy, damaged organelles and proteins undergo lysosomal degradation to supply energy and nutrients to the cell. Moderate autophagy is necessary for embryonic development, and inhibiting autophagy can lead to deformities^17^,^18^. It has been reported that ROS could also elevate the level of autophagy in cells, which could induce cell apoptosis^19^,^20^. The excess ROS induced by high glucose levels could activate autophagy via ER stress signaling^21^.

Currently, more attention is being directed toward studying the effect of maternal hyperglycemia on neural crest development; however, the mechanism for this effect is still unclear. We have previously reported that maternal hyperglycemia could inhibit the neural crest cells that contribute to the dorsal root ganglia^22^. As a classical model for the study of both the cranial neural crest and diabetes, chick embryos have been used to study the effect of high glucose concentrations on embryonic development, and several *in ovo* or shell-less systems have been developed^23^,^24^. In this study, we investigated the cellular and molecular mechanisms of the abnormal development of the cranial neural crest induced by hyperglycemia in the early chick embryo.

## Results

### Exposure to high glucose levels lead to developmental defects in the chick craniofacial skeleton

**Figure 1:**
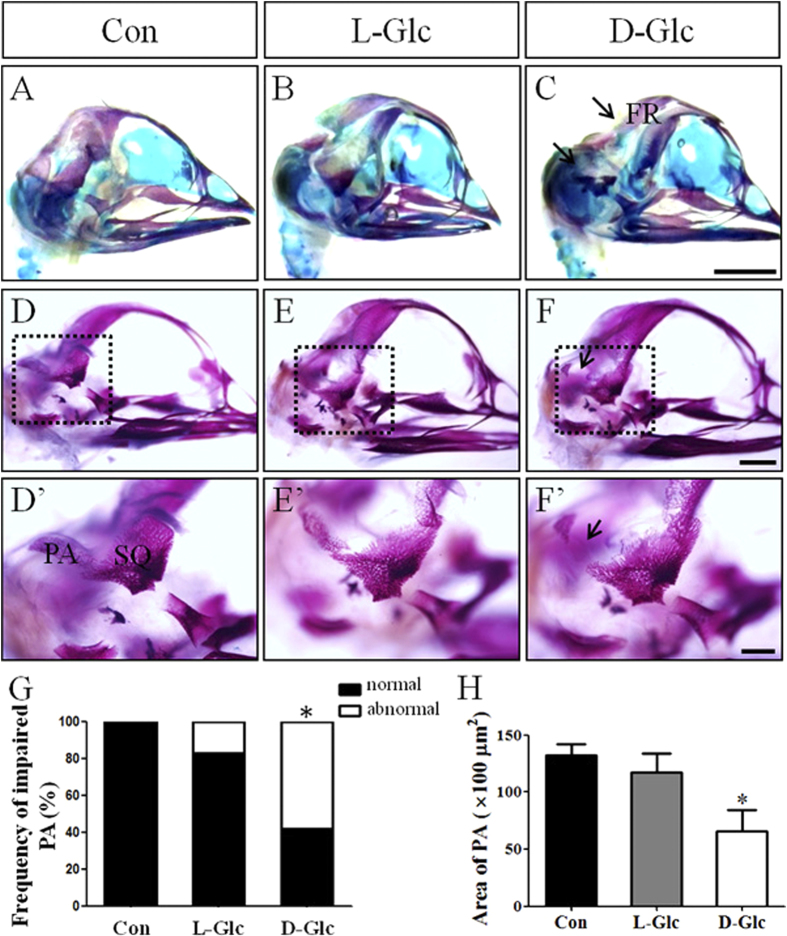
Exposure to high glucose levels causes deficiencies in cranial osteogenesis in chick embryos. (**A–C**) Alcian Blue/Alizarin Red S double staining was performed in control (**A**), 50 mM L-glucose treated (**B**) and 50 mM D-glucose treated (**C**) 12-day chick embryo skulls, respectively. Alcian Blue stains cartilage (blue) and Alizarin Red stains ossified bone (red). We focused on the region of FR (indicated by the black arrows), and PA developmental defects (indicated by the white arrows). (**D–F)** Alizarin Red S staining was performed in control (**D**), 50 mM L-glucose treated (**E**) and 50 mM D-glucose treated (**F**) 12-day chick embryo skulls, respectively. (**D’**–**F’)** The high magnification images from the sites indicated by the black dotted squares in (**D–F**), respectively. Impaired PA was indicated by the black arrows. (**G**) The bar chart showing the frequency comparison of PA developmental defects following exposure to high levels of glucose. (**H**) The bar chart showing the area of PA following glucose treatment. *P < 0.05 indicates a significant difference between the experimental and control embryos. Abbreviations: Con, control; L-Glc, L-Glucose; D-Glc, D-Glucose; PA, parietal bone; FR, frontal bone and SQ, squamous bone. Scale bars = 5 mm in (**A–C**), 2 mm in (**D–F**) and 1 mm in (**D’–F’**).

In our previous study, we demonstrated that the incidence of neural tube defects increased with increasing levels of glucose exposure^22^. Exencephaly might occur if the neural tube defects occur in the cranial neural tube. Of course, not only neurogenesis but also cranial osteogenesis is involved in exencephaly. The craniofacial skeleton of the vertebrate head is a complicated system of interconnected bones and is derived primarily from the cranial neural crest cells. We first examined chondrogenesis and osteogenesis in the chick skull using alcian blue/alizarin red s staining (Fig. 1A–C; N = 10 embryos in each group). In these experiments, we used L-Glucose as osmolarity control for high D-glucose treatment as previously described^24^. The chondrogenesis in the chick skulls in the high glucose-treated group was not different from that in the control group. There appeared to be some developmental defects in the frontal (FR) bones (indicated by the black arrow in Fig. 1B,C) and parietal (PA) bones in the high glucose-treated group (indicated by the white arrow in Fig. 1B,C). Double alcian blue/alizarin red s staining can sometimes obscure the alizarin red s staining in ossified skeletal structures, so the alizarin red s staining was repeated in chick skulls treated with the same high levels of glucose (Fig. 1D–F). In comparison to the control, an obvious defect in parietal (PA) bone development was found, as indicated by the black arrow in Fig. 1F, which could be distinctly observed at high magnification (Fig. 1F’). The incidence of parietal (PA) bone developmental defects was 60% of the total number of chick embryos treated with high levels of D-glucose (Fig. 1G; 0% in control group, N = 0/15 embryos; 18% in L-Glc group, N = 3/17 embryos; and 60% in D-Glc group, N = 12/20 embryos, *P* < 0.05). Additionally, we measured and analyzed the area of parietal bones in both control group and high glucose-treated groups. In presence of glucose, the area of parietal bones decreased obviously (Fig. 1H; Control = 13230 ± 1018 μm^2^; L-Glc = 11780 ± 1618 μm^2^; D-Glc = 6556 ± 1924 μm^2^, *P* < 0.05).

### Exposure to high glucose levels impaired the generation of cranial neural crest cells

**Figure 2:**
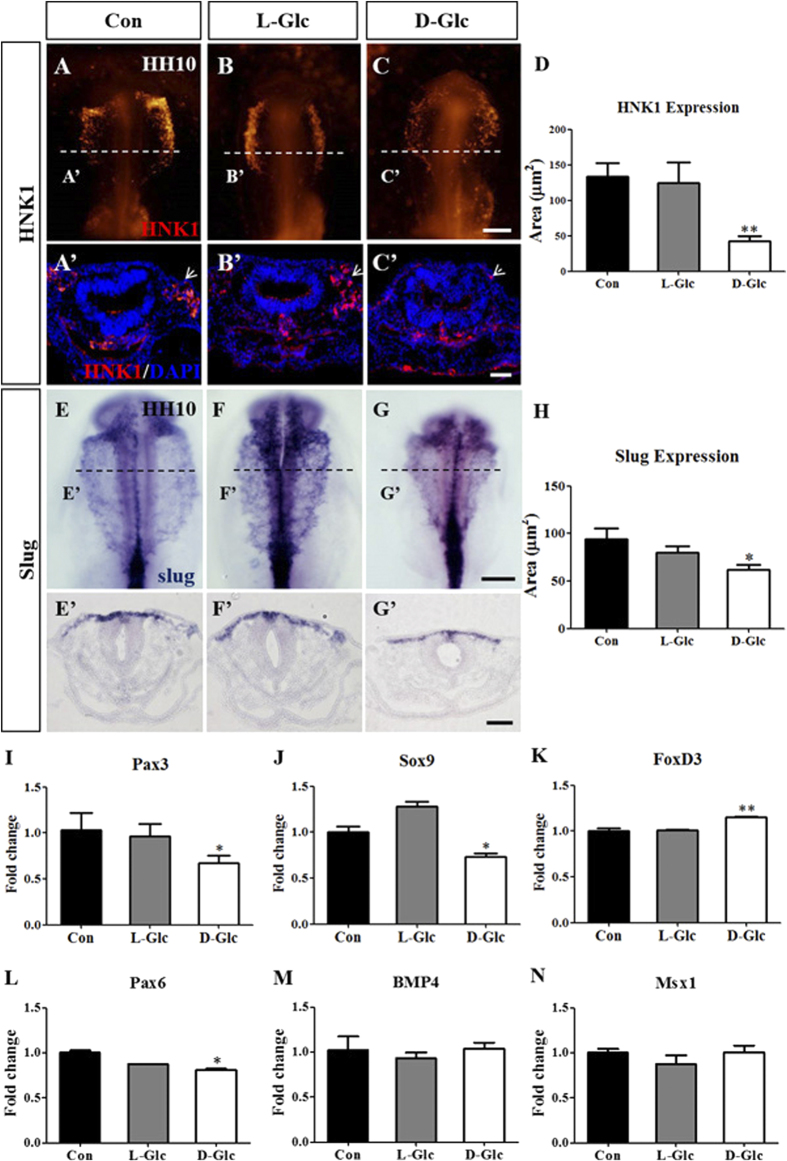
Exposure to high glucose levels restricted the generation of cranial neural crest cells. The chick embryos were exposed to simple saline (control) and high levels of glucose (L-Glucose and D-Glucose) until stage HH10 and harvested for HNK1 immunofluorescent staining and Slug *in situ* hybridization. (**A**–**C)** Immunofluorescent staining against HNK1 was performed in the control and high glucose-treated embryos. The fluorescent images focus on the cranial neural crest cells labeled by HNK1 in the control (**A**), L-Glucose-treated (**B**) and D-Glucose-treated (**C**) embryos. (**A’–C’)** The transverse sections were taken from the levels indicated by the white dotted lines in (**A**–**C)**. DAPI stains in each transverse section. (**G**) The bar chart showing the comparison of cranial HNK1 positive cell areas between the control and high glucose-treated embryos. (**E**–**G)** *In situ* hybridization against Slug was performed in the control and high glucose-treated embryos. The images focus on the cranial neural crest cells labeled by Slug in the control (**E**), L-Glucose-treated (**F**) and D-Glucose-treated (**G**) embryos. (**E’**–**G’**) The transverse sections were taken from the levels indicated by the black dotted lines in (**E–G**). (**H**) The bar chart showing the comparison of Slug positive cranial crest cell areas between the control and high glucose-treated embryos. (**I–N**) Quantitative RT-PCR data showing the expression of Pax3 (**I**), Sox9 (**J**), FoxD3 (**K**), Pax6 (**L**), BMP4 (**M**) and Msx1 (**N**) in cranial neural crest cells from the control and high glucose-treated embryos. *P < 0.05 and **P < 0.01 indicate significant differences between the experimental and control embryos. Abbreviations: Con, control; L-Glc, L-Glucose; D-Glc, D-Glucose. Scale bars = 200 μm in A-C, E-G and 100 μm in A’-C’, E’-G’.

Descendants of the cranial neural crest cells are the primary source of the bones that form the face and the skull^25^. Because we observed a defect in the parietal bones in the presence of high glucose levels, we determined the generation of cranial neural crest cells labeled via HNK1 (migratory neural crest) fluorescent staining following exposure to high glucose levels. The expression of HNK1 in whole-mount chick embryos showed that cranial neural crest cell generation was significantly impaired by D-glucose compared to the control (Fig. 2A–C). We measured the HNK1^+^ area per section on serial transverse sections through the heads of multiple treated embryos (Fig. 2D; Control = 133.2 ± 19.7μm^2^, N = 8 embryos; L-Glc = 124.7 ± 28.5μm^2^, N = 10 embryos; D-Glc = 43.1 ± 7.0μm^2^, N = 10 embryos, *P* = 0.0015), the tendency of HNK1^+^ migratory neural crest cells to be reduced was also shown in the sections (Fig. 2A’–C’) at the levels indicated by the white dotted lines in Fig. 2A–C. Another gene expressed in migratory neural crest cells is Slug^26^. Using *in situ* hybridization to detect Slug in the control and high glucose treated HH10 chick embryos, we found that the production of Slug^+^ migratory cranial neural crest cells was also restricted following the D-glucose treatment (Fig. 2E–G). In the transverse sections of the Slug^+^ *in situ* stained embryos, we are likewise able to demonstrate a reduction in Slug^+^ migratory cranial neural crest cells in the presence of D-glucose (Fig. 2E’–G’). The emigration areas of the Slug^+^ migratory cranial neural crest cells on sections quantifiably reflected this trend (Fig. 2H; Control = 93.9 ± 11.6 μm^2^, N = 7 embryos; L-Glc = 80.1 ± 6.6 μm^2^, N = 7 embryos; D-Glc = 62.3 ± 5.2 μm^2^, N = 8 embryos, *P* = 0.0353). We measured the expression of neural crest markers such as Pax3, Sox9, FoxD3 in the cranial portions of the chick embryos using real-time quantitative PCR following treatment with high levels of glucose. The expression of Pax3 and Sox9 were down-regulated, whereas the expression of FoxD3 was up-regulated (Fig. 2I–K). We also compared the mRNA expression of neural tube markers such as Pax6, BMP4 and EMT marker like Msx1. We found that in presence of high glucose, the expression of Pax6 was inhibited obviously, while, the elevated glucose concentration had no significant effect on the expression of BMP4, Msx1 (Fig. 2L–N). All of these data suggest that exposure to high glucose levels has a negative impact on development of early neural system, especially on the generation of cranial neural crest cells in chick embryos.

### Cell proliferation was not responsible for the reduction of migratory cranial neural crest cells

**Figure 3:**
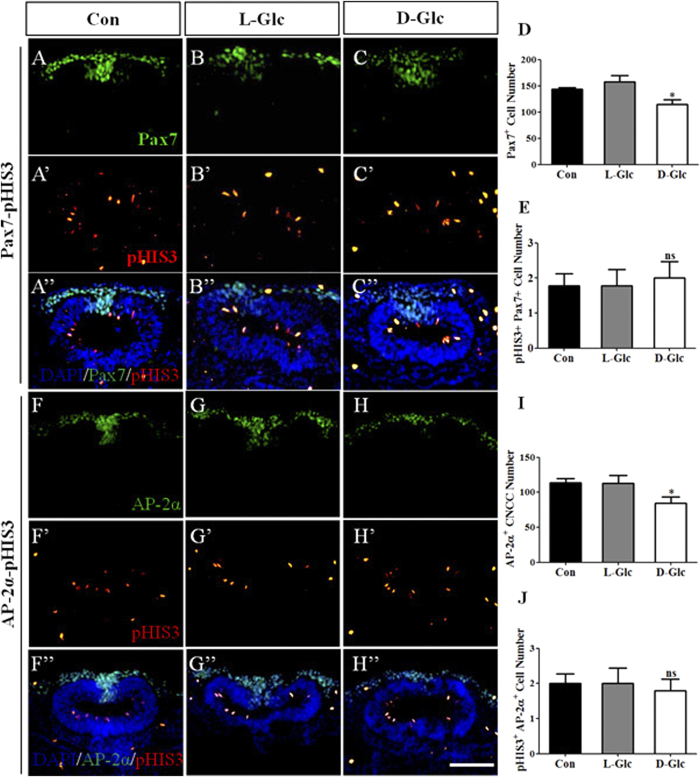
Exposure to high glucose levels did not limit the proliferation of cranial neural crest cells. The chick embryos were exposed to simple saline (control) and high glucose (L-Glucose and D-Glucose) for 48 hours before being harvested for Pax7, AP-2α and pHIS3 immunofluorescent staining. **(A**–**C)** Immunofluorescent staining against Pax7 was performed in the control and high glucose-treated embryos. The fluorescent images of the transverse sections at the same level focus on the cranial neural crest cells and dorsal-side neural tubes labeled by Pax7 in the control (**A**), L-Glucose-treated (**B**) and D-Glucose-treated (**C**) embryos. (**A’-C’**) Immunofluorescent staining against pHIS3 was performed on the same transverse sections as in (**A–C**), respectively. (**A”**–**C”)** The merged images of (**A–C**) and (**A’–C’**) added by DAPI staining, respectively. (**D)** The bar chart showing the comparison of Pax7 positive cranial neural crest cells between the control and high glucose-treated embryos. (**E)** The bar chart showing the comparison of both Pax7 and pHIS3 positive cranial neural crest cells between the control and high glucose-treated embryos. (**F**–**H)** Immunofluorescent staining against AP-2α was performed in the control and high glucose-treated embryos. The fluorescent images of the transverse sections at the same level focus on the migratory cranial neural crest cells labeled by AP-2α in the control (**F**), L-Glucose-treated (**G**) and D-Glucose-treated (**H**) embryos. (**F’–H’**) Immunofluorescent staining against pHIS3 was performed on the same transverse sections as in (**F–H**), respectively. (**F”**–**H”**) The merged images of (**F–H**) and (**F’–H’**) added by DAPI staining, respectively. (**I)** The bar chart showing the comparison of AP-2α positive migratory cranial neural crest cells between the control and high glucose-treated embryos. (**J)** The bar chart showing the comparison of both AP-2α and pHIS3 positive migratory cranial neural crest cells between the control and high glucose-treated embryos. *P < 0.05 and **P < 0.01 indicate significant differences between the experimental and control embryos. Abbreviations: Con, control; L-Glc, L-Glucose; D-Glc, D-Glucose; CNCC, cranial neural crest cell. Scale bars = 100 μm in (**A–H**), (**A’–H’**) and (**A”–H”**).

We wanted to determine whether cell proliferation was associated with the reduction of cranial neural crest cells induced by high glucose treatment. We performed immunofluorescent staining for Pax7 to label both the pre-migratory and migratory neural crest cells (Fig. 3A–C). We measured the Pax7^+^ cell number per section on serial transverse sections through the heads of multiple treated embryos. Again, we found that the production of Pax7^+^ cranial neural crest cells was inhibited by D-glucose in comparison to the control (Fig. 3D; Control = 144 ± 3, L-Glc = 157 ± 12, D-Glc = 114 ± 9, *P* = 0.0177, N = 7 embryos, respectively). Immunofluorescent staining for pHIS3 was carried out simultaneously (Fig. 3A’–C’). We analyzed the number of Pax7^+^-pHIS3^+^ neural crest cells and found that D-glucose exposure did not inhibit the proliferation of cranial neural crest cells (Fig. 3E; Control = 1.8 ± 0.3, L-Glc = 1.8 ± 0.5, D-Glc = 2.0 ± 0.5, *P* = 0.9214, N = 7 embryos, respectively).

AP-2α is expressed only in migratory neural crest cells. Using the same strategy, we carried out double immunofluorescent staining against AP-2α (Fig. 3F–H) and pHIS3 (Fig. 3F’–H’) in HH10 chick embryos. By examining their corresponding transverse sections (Fig. 3F–H), we determined that the number of migratory cranial neural crest cells was reduced in the presence of D-glucose (Fig. 3A”–C”, I; Control = 114 ± 6, N = 6 embryos; L-Glc = 113 ± 11, N = 6 embryos; D-Glc = 84 ± 9, N = 9 embryos, *P* = 0.0172). However, we did not find that exposure to high glucose levels decreased the proliferation of cranial neural crest cells significantly (Fig. 3F”–H”, J; Control = 2 ± 0.3, N = 6 embryos; L-Glc = 2 ± 0.4, N = 6 embryos; D-Glc = 1.8 ± 0.3, N = 9 embryos, *P* = 0.8848) when we carefully analyzed the number of AP-2α^+^-pHIS3^+^ neural crest cells. Our results suggest that the reduction of cranial neural crest cells observed in the presence of high glucose levels might be not due to an inhibitive effect on cell proliferation.

### The apoptosis of the cranial neural crest cells is increased in the presence of high glucose levels

**Figure 4:**
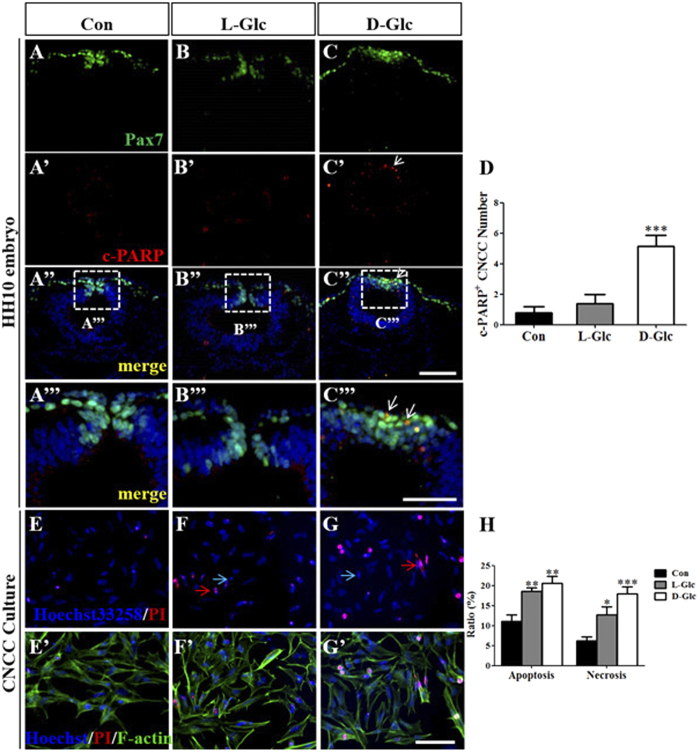
Exposure to high glucose levels increased the levels of apoptosis in the cranial neural crest cells. The chick embryos were exposed to simple saline (control) and high glucose (L-Glucose and D-Glucose) until HH10 before being harvested for Pax7 and c-PARP immunofluorescent staining (**A–C**). **(A–C)** Immunofluorescent staining against Pax7 was performed in the control and high glucose-treated embryos. The fluorescent images of the transverse sections at the same level focus on the cranial neural crest cells and dorsal-side neural tubes labeled by Pax7 in the control (**A**), L-Glucose-treated (**B**) and D-Glucose-treated (**C**) embryos. (**A’–C’**) Immunofluorescent staining against c-PARP was performed on the same transverse sections as shown in (**A–C**), respectively. (**A”-C”)** The merged images of (**A–C**) and (**A’–C’**) added by DAPI staining, respectively. (**A”’–C”’)** The high magnification images from the sites indicated by the white dotted squares in (**A”–C”**), respectively. (**D)** The bar chart showing the comparison of c-PARP positive cranial neural crest cells between the control and high glucose-treated embryos. (**E–G)** Hoechst33258 (blue) **/**Propidium iodide (PI) (red) staining was performed in the primary cultures of cranial neural crest cells (from HH9 chick embryos) in the control (**E**), L-Glucose-treated (**F**) and D-Glucose-treated (**G**) CNCCs. Bright blue apoptotic cells were indicated by blue arrows, and PI^+^ necrotic cells were indicated by red arrows. **(E’–G’)** The merged images of F-actin (green) and (**E–G**), respectively. (**H)** The bar chart comparing the ratio of apoptosis and necrosis between the control and high glucose-treated embryos. *P < 0.05, **P < 0.01 and ***P < 0.001 indicate significant differences between the experimental and control embryos. Abbreviations: Con, control; L-Glc, L-Glucose; D-Glc, D-Glucose; PI, Propidium iodide; CNCC, cranial neural crest cell. Scale bars = 100 μm in (**A–C**), (**A’–C’**), (**A”–H”**); 50 μm in (**A”’–C”’**) and 50 μm in (**E–G**), (**E’-G’**).

High glucose levels did not seem to have a strong effect on cell proliferation, so we considered whether high glucose levels affected the apoptosis because both proliferation and apoptosis are abundant during embryonic development. Pax7 expression was detected using immunofluorescent staining to confirm the cranial neural crest cells (Fig. 4A–C), and c-PARP was employed to determine cell apoptosis in the cranial neural crest cells (Fig. 4A’–C’). Analyzing the merged images of Pax7^+^/c-PARP^+^ (Fig. 4A”–C”, A”’–C”’) showed that the c-PARP^+^ neural crest cells were obviously increased in the presence of D-glucose, which indicates that cell apoptosis was elevated (Fig. 4D; Control = 0.8 ± 0.3, N = 6 embryos; L-Glc = 1.4 ± 0.6, N = 9 embryos; D-Glc = 5 ± 0.7, N = 9 embryos, *P* = 0.0003). We performed double staining for Hoechst and propidium iodide (PI) on primary cultures of cranial neural crest cells to confirm this observation (see the Material and Method in detail) (Fig. 4E,F). F-actin staining was added (Fig. 4E’–G’) to display the neural crest cell morphology. We calculated the ratio between the bright Hoechst cells (apoptotic cell) and the total number of Hoechst positive cells and the ratio between cells that were strongly stained with PI and the total number of Hoechst positive cells to distinguish apoptotic from necrotic cells. The results showed that both apoptosis and necrosis were elevated in the presence of high glucose levels (Fig. 4H; Apoptotic cells of total: Control = 11.07 ± 1.676%, L-Glc = 18.53 ± 0.9128%, D-Glc = 20.59 ± 1.701%, *P* = 0.0015; Necrotic cells of total: Control = 6.216 ± 1.040%, L-Glc = 12.66 ± 2.017%, D-Glc = 18.00 ± 1.672%, *P* = 0.0011; Control: N = 6 explants, L-Glc: N = 7 explants, D-Glc: N = 8 explants). To explore the involved mechanisms in cranial neural crest cell apoptosis, we employed Pifithrin-μ, which can specifically inhibit P53-dependent mitochondrial pathway of apoptosis. With the same strategy, we found that the c-PARP^+^ neural crest cells were obviously decreased in the presence of Pfμ (Supplementary Figure-1A-H, *P* < 0.001), which indicates that cell apoptosis was induced by D-glucose in a P53-dependent manner. Additionally, using HEK293 cell, we tried to find a common mechanism of cell death induced by glucose. The HEK293 cell line was employed to detect the protein expression of apoptosis-related genes using Western blot. We demonstrated that high glucose treatment increased the expression of P53, pro-Cas7, Cleaved-Cas7, pro-Cas3 and Cleaved-Cas3. These results suggested that elevated glucose levels could trigger cell apoptosis through a P53-dependent manner (Supplementary Figure-1I). We propose that apoptosis is involved in the inhibition of cranial neural crest cells that is induced by exposure to high glucose levels.

### Exposure to high glucose levels elevated the expression of autophagy-associated genes in neural crest and HEK293 cells

**Figure 5:**
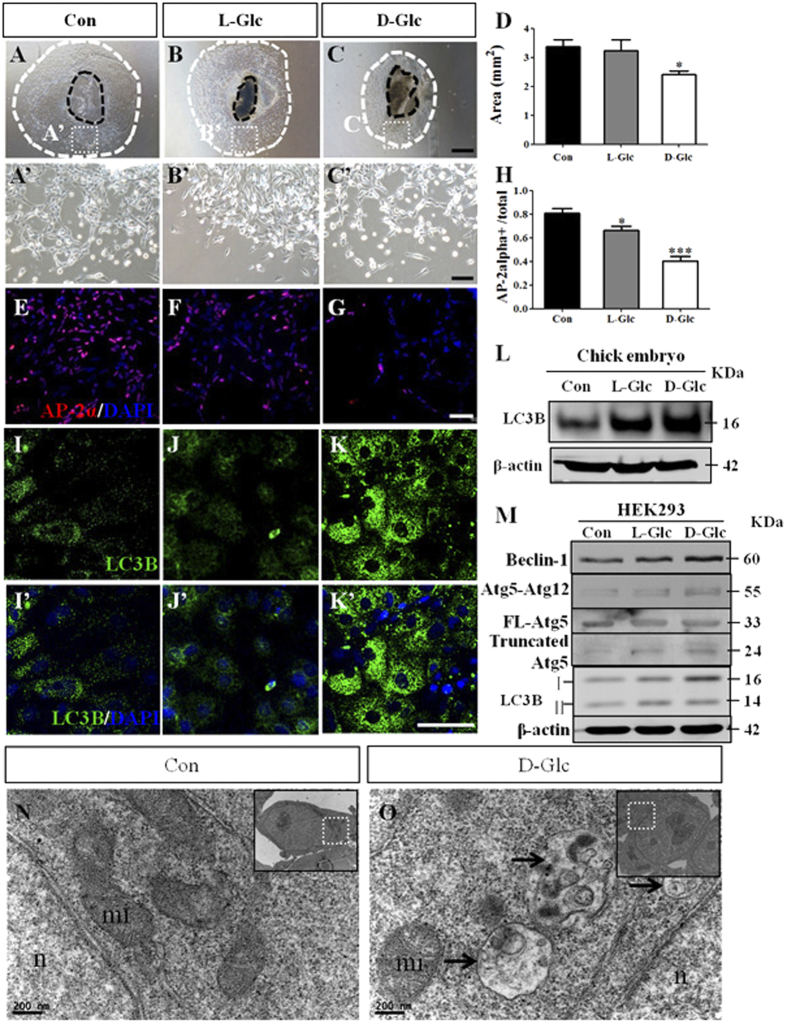
The alteration of autophagy gene expression when high glucose inhibited the generation of cranial neural crest cells. HH9 chick embryonic neural tube tissues were incubated *in vitro* for 48 hours. (**A-C)** The bright-field images of the CNCCs that emigrated from the primary cultures of neural tubes in the presence of simple saline. Control, (**A**), L-Glucose (**B**) and D-Glucose (**C**), respectively. (**A’–C’)** The high magnification images from the sites indicated by the white dotted squares in (**A–C**), respectively. (**D)** The bar chart showing the comparison of the migratory cell areas from the cultured neural tubes between the control and high glucose-treated embryos. (**E–G)** Immunofluorescent staining against AP-2α was performed on the primary cultures as shown in **(A–C**), respectively. The merged images of AP-2α staining + DAPI were taken at the same position relative to the neural tubes (**A–C**) in the control (**E**), L-Glucose (**F**) and D-Glucose (**G**) treated primary cultures. (**H)** The bar chart showing the comparison of AP-2α positive cranial neural crest cell numbers among control and high glucose-treated embryos. (**I–K)** Immunofluorescent staining against LC3B was performed on the primary cultures of neural tube as shown in (A**–**C), respectively. The fluorescent images of LC3B were taken at the same position related to the neural tubes in the control (**I**), L-Glucose (**J**) and D-Glucose (**K**) treated primary cultures. (**I’–K’)** The merged images of I-K + DAPI, respectively. (**L)** Western blots showing the expression of LC3B in the control and high glucose-treated embryos. (**M)** Western blots showing the protein expression of Beclin1, Atg5-Atg12, FL-Atg5, Truncated Atg5 and LC3B in HEK293 cells in the presence of simple saline (control) and high glucose. (**N–O**) Transmission electron microscopy of the control and D-Glucose treated HH10 chick cranial neural crest cells, the autophagosomes are indicated by the black arrowheads. *P < 0.05, **P < 0.01 and ***P < 0.001 indicate significant differences between the experimental and control embryos. Abbreviations: Con, control; L-Glc, L-Glucose; D-Glc, D-Glucose; PI, Propidium iodide; CNCC, cranial neural crest cell; n: nucleus; mi: mitochondrion. Scale bars = 500 μm in (**A–C**); 100 μm in (**A’–C’**), 50 μm in E-G and 50 μm in (**I–K**), (**I’–K’**), 200 nm in (**N–O**).

Cell apoptosis might play an important role in the reduction of the cranial neural crest cells induced by high glucose levels, and dysfunctional autophagy can cause cell apoptosis^27^. Therefore, we wanted to study whether autophagy is involved in the inhibitory effect. Primary cultures of cranial neural crest cells were used because it is easier to detect autophagy-associated gene expression in cell cultures than in embryo samples. We observed that the emigration of neural crest cells from neural tube tissues in the presence of D-glucose was reduced compared to the control (Fig. 5A–C), and the area statistics of neural crest cell emigration showed the same trend (Fig. 5D; Control = 3.4 ± 0.4 mm^2^, N = 10 explants; L-Glc = 3.2 ± 0.6 mm^2^, N = 10 explants, D-Glc = 2.4 ± 0.1 mm^2^, N = 12 explants; *P* = 0.0312), confirming the inhibitory effect of high glucose levels on the generation of neural crest cells. In addition, bright/round cells were increased in the D-glucose treated group, indicating that there are more dead cells in the presence of high glucose levels. AP-2α^+^ cells were decreased when the primary cultures of neural crest cells were exposed to high glucose levels (Fig. 5E–H, AP-2α^+^ cells versus total cells: Control = 81.05 ± 3.988%, L-Glc = 66.56 ± 3.124%, D-Glc = 40.57 ± 3.762%; N = 6 explants in each group, *P* < *0.001*). Our observations in primary cultures of neural crest cells cultured in the presence of high glucose levels are similar to the *in vivo* embryo experiments. We determined the LC3B expression because LC3B is an important gene in activating autophagy and found elevated LC3B expression and autophagosome-like particles around the cell nuclei in the presence of D-glucose compared to the control (Fig. 5I–K, I’–K’). Furthermore, Western blots also showed that the high glucose treatment enhanced LC3B protein expression in the chick embryos (Fig. 5L). In addition, we exposed HEK293 cells to high levels of glucose and performed Western blots to detect autophagy-related gene expression. We found increased expression of Beclin-1 and Atg5-Atg12, slightly decreased expression of FL-Atg5, and elevated expression of truncated Atg5. Both LC3B I and LC3B II expression rose (Fig. 5M). We employed transmission electron microscopy (TEM) to observe the autophagosomes in HH10 chick cranial crest cells to verify that autophagy was induced by elevated glucose levels. Compared to the control, we found more autophagosomes in the D-glucose treated cells (Fig. 5N–O). Atg5 cleavage mediated by Calpain is a well-known cell type-independent switch between autophagy and apoptosis^20^. In summary, autophagy is activated in the presence of high glucose treatment, implying that it might play role on the reduction of cranial neural crest cells partly through truncated Atg5.

### Functional evidence for the involvement of autophagy in the reduction of neural crest cells induced by high glucose levels

**Figure 6:**
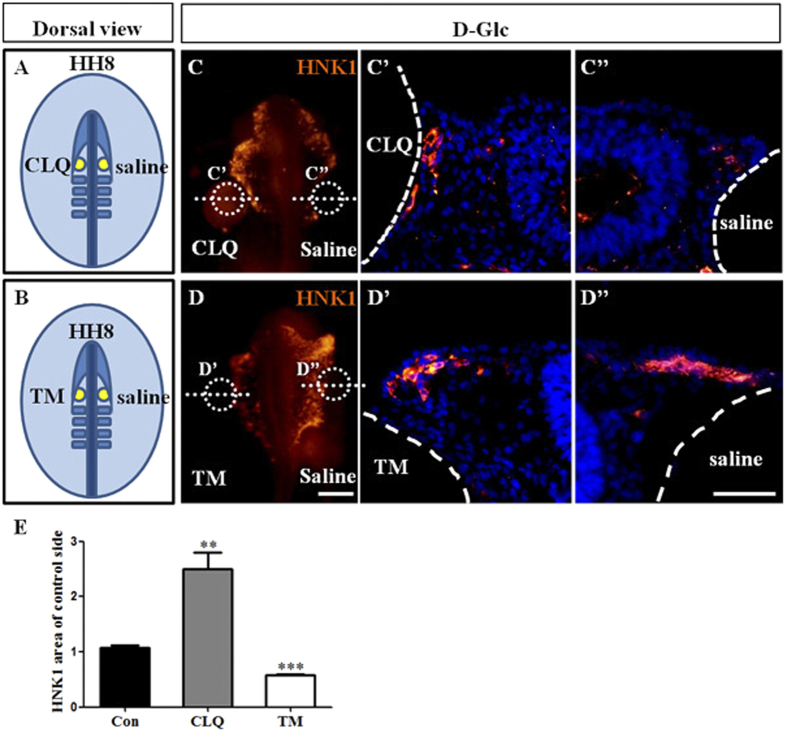
Both the stimulation and inhibition of autophagy affected the generation of cranial neural crest cells. Heparin beads soaked with simple saline (right) and either 50 μM CLQ or 4 μg/ml TM (left) were implanted into the head folds of HH8 chick embryos by pushing them as schematically shown in (**A,B)** and the chick embryos were incubated to HH10. The simple saline beads were used as the control to exclude the variability in individual embryo development. (**C,D)** Immunofluorescent staining against HNK1 was performed on the chick embryos. The positions of the CLQ, TM and simple saline beads are indicated by the white dotted circles in (**C,D**), respectively. (**C’–C”)** The transverse sections of the chick embryo at the levels indicated by the white dotted lines in (**C**). (**C’**) is near to the CLQ beads while (**C”**) is near to the simple saline beads. (**D’–D”)** The transverse sections of the chick embryos at the levels indicated by the white dotted lines in (**D**). (**D’**) is near to the TM beads while (**D”**) is near to the simple saline beads. (**E)** The bar chart showing the ratio comparison of cranial neural crest cell areas between the sides with either the CLQ or TM beads and the side with the control beads. Abbreviations: Con, control; D-Glc, D-Glucose; CLQ, Hydroxychloroquine; TM, tunicamycin. Scale bars = 200 μm in (**C–D**) and 100 μm in (**C’–C”, D’–D”**).

We demonstrated that there were changes in autophagy-associated gene expression when embryos or cells exposed to high glucose. We then investigated whether modulating autophagy in the presence of high glucose could directly affect the production of cranial neural crest cells using either Hydroxychloroquine (CLQ, autophagy inhibitor) or tunicamycin (TM, autophagy agonist)^28^ as schematically shown in Fig. 6A,B. Either CLQ or TM soaked beads were implanted in the head folds, and simple saline soaked beads were implanted on the opposite side as a control (Fig. 6A,B). The HNK1 immunofluorescent staining was performed to label neural crest cells as the embryos developed until HH10. The result showed that more cranial neural crest cells were produced when autophagy was inhibited with CLQ beads in comparison to the control beads (Fig. 6C; HNK1 area of control side: Con = 1.1 ± 0.05, N = 5 embryos; CLQ = 2.5 ± 0.3, N = 6 embryos, *P* = 0.0021). This could be clearly observed in the transverse sections (Fig. 6C’–C”; HNK1). On the contrary, fewer neural crest cells were produced when autophagy was activated with TM beads (Fig. 6D; HNK1 area of control side: Con = 1.1 ± 0.05, N = 5 embryos; TM = 0.6 ± 0.02, N = 6 embryos, *P* < 0.001). This could also be observed in the transverse sections (Fig. 6D’–D”). This verified that autophagy activation could inhibit neural crest production while autophagy inhibition could increase neural crest production in the presence of elevated glucose (Fig. 6E).

### High glucose-induced autophagy is dependent on the ERK rather than the mTOR pathway

**Figure 7:**
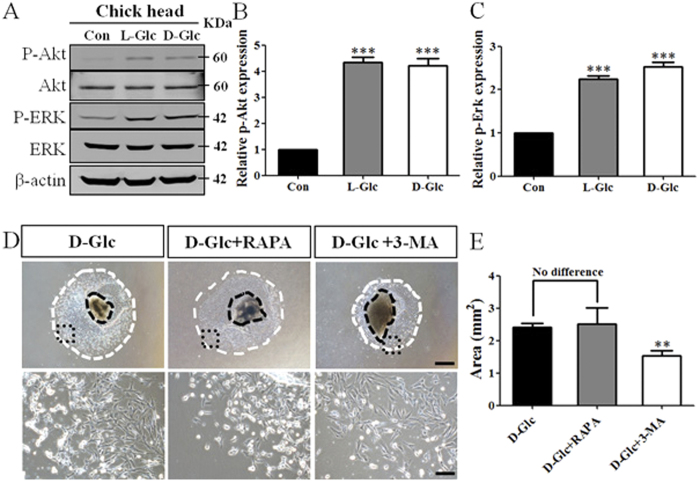
The ERK pathway plays a more important role than the Akt-mTOR pathway in the autophagy induced by elevated glucose levels. The expression and phosphorylation of Akt and Erk were measured at the protein level in the control and glucose-treated HH10 chick heads. (**A**) Western blots showing the expression of phospho-Akt (p-Akt), total Akt, phosphor-Erk (p-Erk) and total Erk. (**B,C**) The relative expression of p-Akt and p-Erk compared to β-actin was measured. (**D**) HH9 chick embryonic neural tube tissues of the same size were incubated *in vitro* in the presence of either D-Glucose and D-Glucose with Rapamycin (mTOR inhibitor) or 3-MA (class III PI3K inhibitor), respectively. The upper panel displays bright-field images of the cranial neural crest cells that emigrated from the 48-hour primary cultures of neural tubes. The lower panel displays high magnification images from the sites indicated by the black dotted squares in the corresponding upper panel images, respectively. (**E)** The bar chart showing the comparison of migratory neural crest areas from the 48-hour cultured neural tubes between the D-Glc and D-Glc + 200 nM Rapamycin and D-Glc + 5 mM 3-MA treated groups (n = 6 in each group). (**G)** The bar chart showing the ratio of p-AKT/Akt protein expression in HEK293 cells between the control and high glucose-treated groups. ***P < 0.001 and **P < 0.01 indicate significant differences between the experimental and control embryos. Abbreviations: Con, control; L-Glc, L-Glucose; D-Glc, D-Glucose; RAPA: Rapamycin; 3-MA: 3-methyladenine. Scale bars = 500 μm in the upper panel and 100 μm in the lower panel of (**D**).

The PI3K/Akt/mTOR pathway is known to play a very important role in the autophagy process^29^. The activation of the MEK/ERK pathway also has a critical impact on cytoprotective autophagy^30^. Therefore, we wanted to determine whether PI3K/Akt and ERK signaling are involved in the regulatory mechanism. We determined the levels of protein expression of phosphorylated (p-) Akt and Akt, p-ERK, and ERK in chick head cells in response to high glucose treatment by performing Western blots. Glucose distinctly up-regulated p-Akt and p-ERK expression (Fig. 7A). The relative expression of p-Akt and p-ERK to β-actin was measured (Fig. 7B–C, *P* < 0.001), suggesting that the ERK signaling could be activated due to high glucose exposure. The activation of Akt indicated that PI3K/Akt signaling was activated, and which could be an upstream regulator of mTOR. We exposed primary cultures of cranial neural crest cells to high glucose levels and either Rapamycin, an inhibitor of mTOR, or 3-MA, a type III PI3K inhibitor, for 48 hours *in vitro* to investigate the direct role of mTOR in elevated glucose-induced autophagy^28^. We found that the emigration of neural crest cells from neural tube tissues in the presence of either D-glucose or D-glucose with Rapamycin were similar, indicating the inhibition of mTOR had no effect on neural crest abundance. Nevertheless, the emigration of neural crest cells exposed to D-glucose and 3-MA was obviously reduced compared to the D-glucose group (Fig. 7D upper panel), and the area statistics of the neural crest cell emigration reflected this trend (Fig. 7E; D-Glc = 2.4 ± 0.1 mm^2^, N = 12 explants; D-Glc + RAPA = 2.5 ± 0.5 mm^2^, N = 6 explants, *P* = 0.7836; D-Glc + 3-MA = 1.5 ± 0.2 mm^2^, N = 9 explants, *P* = 0.0002). In addition, we observed that D-glucose and 3-MA significantly suppressed the abundance of neural crest cells that detached from the cultured cranial neural tubes *in vitro* (Fig. 7D lower panel), suggesting that the inhibition of PI3K signaling impaired cell survival during treatment with high levels of glucose. Taken together, the results suggest that ERK signaling rather than the mTOR pathway is the major signaling pathway involved in elevated glucose-induced autophagy.

## Discussions

High glucose environment is associated with a high risk of fetal malformation. However, very little is known about the influence of hyperglycemia on cranial neural crest cell production. In this study, we found defects in partial bone development following treatment with high levels of glucose, especially treatment with D-glucose (Fig. 1G). L-glucose exposure also causes parietal bone defects in a few cases. This effect might be due to high glucose-induced osmotic pressure, which also occurred in our high salt-exposure experiments^31^. However, under normal circumstances, osmolarity is stable in the human body. The osteogenesis of the skull is completed through intramembranous ossification. The cranial neural crest cells are the principal components that contribute to the mesenchymal cells, where ossification will occur 25. Therefore, we investigated the early stage of cranial neural crest cell production in the experiments described here.

HNK1 is expressed in migratory neural crest cells and was employed to label the migratory cranial neural crest cells. We found that D-glucose significantly inhibits cranial neural crest cell abundance (Fig. 2A–D). And the production of Slug^+^ cranial neural crest cells demonstrated the same trend (Fig. 2E–H) as was observed using HNK1. We also measured the expression of Pax3, Sox9, FoxD3, Pax6, BMP4 and Msx1, which are also associated with the generation of the neural crest: Pax3, Pax6 and Sox9 expression were decreased, but FoxD3 expression was increased following exposure to high glucose levels, while, the elevated glucose concentration had no significant effect on the expression of BMP4, Msx1 (Fig. 2I–N). This might suggest that high glucose treatment leads to a reduction in cranial neural crest cells by influencing the expression of Slug, Pax3 and Sox9. Our current observations are similar to the findings of Wentzel that Pax3 mRNA levels were inhibited by high glucose exposure in both cranial and trunk neural crest explant cultures^13^.

The next question we addressed was how the cranial neural crest cell production was restricted by exposure to high glucose levels. Neural crest cells possess some stem cell characteristics; they continue proliferating and differentiating while migrating to their final destination. Thus, we addressed whether the proliferation of cranial neural crest cells was affected by exposure to high levels of glucose. Because Pax7 could label the migratory neural crest cells and the dorsal side of the neural tube, we enumerated the proliferating cranial neural crest cells (Pax7^+^- pHIS3^+^ cells) (Fig. 3A–E). We found that high glucose levels caused a reduction in the number of detached cranial neural crest cells; similar results are obtained using the migratory cranial neural crest marker, AP-2α (Fig. 3F–I). However, it is rather remarkable that the number of both AP-2α^+^ and pHIS3^+^ neural crest cells were similar to the controls (Fig. 3J), suggesting that migratory cranial neural crest cell proliferation is not affected by high glucose treatment. We next examined the possibility that the presence of high glucose levels induces apoptosis in cranial neural crest cells.

Again, Pax7 was employed to label the cranial neural crest cells (Fig. 4A–C), and cleaved-PARP (c-PARP, cell apotosis marker) was used to detect apoptotic cells (Fig. 4A’–C’). The number of c-PARP^+^-Pax7^+^ cells increased in the presence of high levels of glucose. This result demonstrated that cranial neural crest cells undergo more apoptosis following treatment with high levels of glucose (Fig. 4D). Cell death consists of apoptosis and necrosis. Propidium iodide strongly stains dead cells and we analyzed propidium iodide and Hoechst33258 double positive cells in primary cultures of neural crest cells to determine whether necrosis was also elevated in the presence of high glucose levels (Fig. 4E–G,H). Coincidentally, Cederberg *et al.* (2003) reported that high-glucose exposure-induced cell death of neural crest derived cells was responsible for the developmental disorder of neural crest-derived structures in diabetic rats embryos^8^. Utilizing specific P53 inhibitor, Pifithrin-μ, we found that elevated glucose concentration could induce apoptosis in a P53-dependent manner. At the meantime, we compared the Pax7^+^ cell number between D-glucose and D-glucose combined with Pfμ treated groups. The Pax7^+^ cells decreased significantly in the D-glucose combined with Pfμ treated group (Supplementary Figure-1G, *P* < 0.05). P53 plays a critical and complex role in regulating apoptosis, necrosis and autophagy, which can promote either cell death or cell survival under stress. Activated P53 in nuclear can induce autophagy. When apoptosis is compromised, autophagy can promote cell death^32^,^33^. To further understand the mechanisms of high glucose induced cell death, more effort is required.

We next addressed the cause of the elevated levels of cell death among the cranial neural crest cells that were exposed to high levels of glucose. In our previous studies, we showed that high glucose treatment could cause excessive ROS generation^22^ and that the excess ROS might in turn damage the cells through dysfunctional autophagy^34^. Hence, we measured the expression of the autophagy-associated gene, LC3B, in primary cultures of cranial neural crest cells after verifying the failure of AP-2α^+^ neural crest cells to detach from cultured neural tubes in the presence of high levels of glucose (Fig. 5A–H). Following treatment with high levels of glucose, we detected elevated LC3B expression via both immunofluorescent staining and Western blot assays (Fig. 5I–L). To further confirm the correlation between autophagy and high glucose treatment, we exposed HEK293 cells to high glucose and used western blot to show that the expression of Beclin-1 and LC3B II were elevated and that Atg5-Atg12 complexes were increased, implying autophagy activation. Truncated-Atg5 expression was increased while FL-Atg5 expression was decreased slightly. Calpain mediated Atg5 cleavage is a cell type-independent switch between autophagy and apoptosis^20^. The expression of autophagy-associated genes suggests the activation of autophagy in the presence of high levels of glucose, and the excess autophagy induced by high glucose provoked cell apoptosis via truncated Atg5. The experiments in which CLQ or TM beads were implanted were designed to verify the functional role of autophagy in the cranial neural crest (Fig. 6A,B). As might be expected, we found that the inhibition of autophagy with CLQ could enhance the production of cranial neural crest cells (Fig. 6C–C”, E), whereas the activation of autophagy with TM lessened the production of cranial neural crest cells (Fig. 7D–D”, E), indicating that there is a functional correlation between autophagy and high glucose exposure. These results are in accordance with the report by Adastra, KL *et al.*^16^, in which they identified a variety of pathways for activating autophagy in mouse embryos and oocytes in response to a hyperglycemic environment.

**Figure 8:**
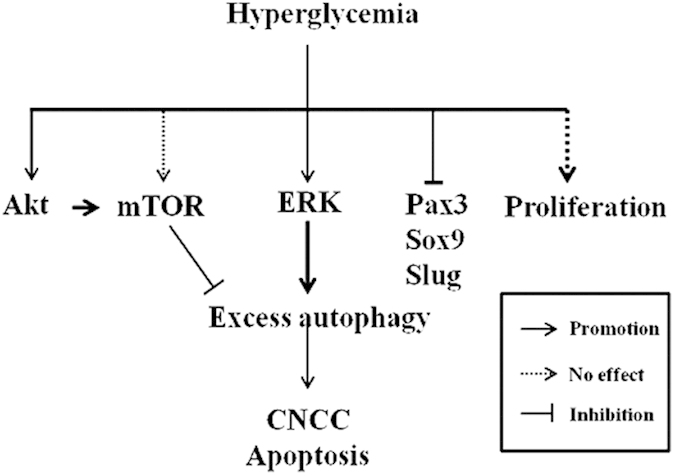
A proposed model that depicts the potential mechanisms by which high glucose exposure leads to defects in cranial neural crest cell generation. High levels of glucose might trigger autophagic disturbances and promote ERK activation. The expression of the neural crest development-related genes Pax3, Sox9 and Slug were also adversely influenced. It is well known that elevated levels of glucose can also induce autophagy through ER stress, although we did not address this mechanism in the current study. In summary, we propose that excessive cell autophagy induced by high glucose levels is the key factor that induces increased apoptosis in the cranial neural crest.

Altering either the external or the cellular environment could activate autophagy through different signaling pathways. The MEK/ERK and PI3K/Akt/mTOR signaling pathways play vital roles in regulating cellular autophagy^29^,^35^, and Class III PI3K plays an essential role in the initiation of autophagosome formation^36^. When we measured the phosphorylation of Akt and ERK in HH10 chick heads, the up-regulation of p-Akt and p-ERK indicated the activation of PI3K/Akt and ERK signaling by exposure to high glucose levels (Fig. 7A–C). We combined the specific inhibitor of mTOR, Rapamycin, with D-glucose in primary cultures of cranial neural crest cells and found that the production of cranial neural crest cells was not significantly affected compared to the D-glucose group. The survival of neural crest cells was significantly inhibited by D-glucose with 3-MA (Fig. 7D,E). These results suggest that the activation of autophagy induced by high glucose is independent of mTOR and that PI3K/Akt signaling possibly plays a key role in cell survival in the presence of high glucose levels. In summary, our current experimental data suggest that exposure to high glucose levels leads to excessive autophagy in the cranial neural crest cells through an mTOR independent pathway. ERK signaling could be a major pathway involved in this. Furthermore, the excess autophagy could lead to cell apoptosis in cranial neural crest cells, ultimately reducing the production of cranial neural crest cells in the presence of high levels of glucose (Fig. 8). Further experiments are certainly required to determine the regulatory mechanism at the molecular level. There is no doubt that the more we understand about the pathological and molecular mechanisms underlying cranial neural crest cell dysplasia in response to high glucose levels, the better we will be able to prevent and treat NCC diseases (neurocristopathies).

## Methods

### Chick embryos and Glucose application

Fertilized leghorn eggs were acquired from the Avian Farm of the South China Agriculture University (Guangzhou, China). Early developing chick embryos are not considered to be animals, so serious ethical issues are not evoked^37^. The fertilized eggs were incubated in a humidified incubator (Yiheng Instruments, Shanghai, China) at 38 °C and 70% humidity until the desired Hamburger-Hamilton (HH) stage of chick embryo development was reached^38^. The glucose treatment *in ovo* was performed according to the methods described by Hinds group^24^. Before experimentation, the air chamber of the eggs was marked under an egg candler. The shell above the air chamber was carefully removed, and 50 μl of 50 mM D-Glucose (dissolved in 0.72% sodium chloride, Sigma, G7528), L-Glucose (osmolarity control, Sigma, G5500) or chick simple saline (0.72% sodium chloride) was injected into the air chamber of the chick embryos every day from 0-day until 12-day. The experiments were performed in triplicate with 10 eggs in each group, and the surviving embryos were harvested for skeleton staining.

For early chick embryos, the same methods for treatment in early chick (EC)^39^ cultures as those we described previously were employed in this study^18^. Briefly, HH1 embryos were exposed to either D-glucose or L-glucose containing medium (90 mg glucose/10 ml medium) and then incubated at 37 °C with humidity until HH10. To understand the role of P53 in apoptosis induced by high glucose, EC cultured embryos were treated with D-glucose combined with Pifithrin-μ (20μM, SIGMA, #P0122) until HH10. Embryos exposed to D-Glucose only were set as control.

### Alcian blue/alizarin red staining of whole embryos

The 12-day (E12) chick embryos mentioned above were stained with alcian blue and alizarin red dyes to visualize the craniofacial skeleton, as previously described^40^. Briefly, the embryos were fixed in 95% ethanol for 2 hours and then the skin and viscera were carefully removed and post-fixed for 1 day. Next, the embryos were stained in 0.1% alcian blue and/or alizarin red (Solarbio, Beijing, China) dyes in 70% ethanol for 1 day and then cleared in 25% glycerol/0.5% KOH for 3 days. Finally, the embryos were treated in a graded series of glycerol solutions. Then, the cranial skeleton was photographed using a stereomicroscope (Olympus, MVX10).

### Whole-mount Embryo Immunostaining

Whole-mount immunostaining of chick embryos was performed to detect corresponding protein expression as previously described^41^.The chick embryos were harvested after incubation and fixed in 4% PFA overnight at 4 °C. The whole-mount embryo immunostaining was performed using the following antibodies: phospho-Histone3 (pHIS3; 1:400, Santa Cruz, sc-12927-R), Pax7 (1:100, DSHB), AP-2α (1:100, DSHB), HNK-1 (1:500, Sigma, F6148), cleaved-PARP (1:100, Cell Signaling Technology, #9915) and LC3B (1:100, Cell Signaling Technology, #3808). Briefly, the fixed chick embryos were incubated with the primary antibody at 4 °C overnight on a shaker. After extensive rinsing in PBST (0.1% Tween-20), the embryos were incubated with the corresponding alexa fluor 555 or 488 secondary antibody (1:1000, Invitrogen, A-11029, A-21426, A-11008) at 4 °C overnight on a shaker. For double staining, the samples were incubated consecutively with one antibody at a time. All the embryos were counterstained with DAPI (1:1000, Invitrogen, D1306) at room temperature for 1 hour. Subsequently, the embryos sliced into 10 μm sections using a cryostat apparatus (Leica CM1900).

### *In situ* hybridization

The whole-mount *in situ* hybridization of the chick embryos was performed according to the standard *in situ* hybridization protocol^42^. Digoxigenin-labeled probes were synthesized against *Slug.* The whole-mount stained embryos were photographed, and then, frozen sections were prepared from them by sectioning at thickness of 16 μm.

### Transmission electron microscopy

The control and glucose treated HH10 chick embryos were fixed with 2.5% glutaral in 0.1 M PBS for 2 h, and then, the chick heads were dissected. The samples were sent to the TEM Laboratory of Sun Yat-sen University. The embedding, ultrathin sectioning and staining were performed by professional technicians and examined using a Tecnai G^2^ Spirit Twin (FEI, USA). The observation of autophagosomes using TEM was according to the guidelines for monitoring autophagy^28^.

### RNA isolation and Quantitative PCR

The total RNA was isolated from the HH10 chick heads (N > 20 embryos in each group) using a Trizol kit (Invitrogen, #15596018) according to the manufacturer’s instructions. The first-strand cDNA synthesis and the SYBR^®^ Green qPCR assay were performed using the PrimeScript^TM^ RT reagent kit (Takara, Japan) and specific primers as follows: PPIA:5′-TGACAAGGTGCCCATAACAG -3′, 5′-TTCTCGTCGGCAAACTTCTC-3′^24^; Pax3:5′-TGGAGCCCACCACCACTGTC-3′, 5′-AACACCAGCTTAACTTGAAG-3′; Sox9:5′-ACCAACCACGCAGGAGGAAG-3′, 5′-TCGCTGATGCTGGAGGATGAC-3′; FoxD3:5′-GCAGAGCCCGCAGAAGAAGC-3′, 5′-CGTTGAGCGAGAGGTTGTGG-3′; Pax6:5′-GCCAAGTGGAGAAGGGAGGAGAAG-3′, 5′-GCTGGTAAACGCTTGTGCTGAAAC-3′; BMP4:5′-CAACTCCACCAACCACGCCATC-3′, 5′-CAGCACCACCTTGTCATACTCATCC-3′; Msx1:5′-AGACTTCTCCGCTCCCTTCATCC-3′, 5′-TGCCTTTGTGCCCTTTCTCTGC-3′^43^. The reverse transcription and amplification reactions were performed in Bio-Rad S1000^TM^ and ABI 7000 thermal cyclers, respectively. The expression of the genes was normalized to the housekeeping gene *PPIA*, and the expression level was compared by ΔΔCt^44^.

### Primary Culture of NCCs and cell staining

The NCCs were prepared from the neural tube (at head level) of the chick embryos, according to the methods previously described^45^. Briefly, the fertilized chick eggs were incubated until the 7-9 somite stage (HH 9-9+). The neural tubes were dissected from the head of the embryos and explanted into 3.5 mm dishes in DMEM (100 μl/dish, containing 5.5 mM D-glucose, Life, #11885-092) for 6 hours at 37 °C and 5% CO_2_ to allow the explants to adhere. A few of the NCCs migrated from the neural tubes after the incubation and then 1 ml of medium containing 50 mM D-glucose/L-glucose or with 200 nM Rapamycin (dissolved in DMSO, <0.1%, final concentration) or 5 mM 3-Methyladenine (3-MA, dissolved in simple saline) was introduced into the cultures for 48 hours. The migration area and was measured using Image-Pro Plus 7.0 software as we described previously^46^.

To determine apoptosis and cell death, the Hoechst/Propidium Iodide (PI) double staining experiments were carried out according to the methods described previously^47^. Briefly, after incubation, the culture medium was removed, and the neural crest cells were rinsed with pre-warmed PBS and stained in 1 ml of PBS containing 10 μg/ml Hoechst33258 (Life, H1398) and 10 μg/ml PI (Life, P1304MP) at 37 °C for 15 min. After staining, the cultures were washed thoroughly with PBS and fixed with 4% paraformaldehyde at room temperature for 15 min. Following a wash, F-actin (1:1000, CST, #8878) staining was performed at 4 °C overnight. Then, images were taken using an Olympus IX51epi-fluorescent microscope (340 nm, 488 nm and 620 nm respectively). We randomly selected six visual fields (at 400X) from each explant and manually counted the PI^+^Hoechst^+^ cells or bright blue Hoechst^+^ cells versus total Hoechst^+^ cells using Image-Pro Plus 7.0 software.

### Bead experiments

The bead experiments were performed according to the methods described by Yamada group previously^43^. Briefly, heparin beads were soaked in Hydroxychloroquine (CLQ, 50 μM, autophagy inhibitor, Sigma, H0915) or tunicamycin (TM, 4 μg/ml, autophagy agonist, Millipore, #654380)^28^ for 2 h and then implanted into the cranial region of HH8 chick embryos in EC culture, by the side of the head neural tube. The beads soaked in simple saline were used as a control (as shown in Fig. 6A,C). The embryos were continually incubated until HH10. After section, we manually quantified the HNK1^+^ area of treated side versus control side with Image-Pro Plus 7.0 software.

### Cell line culture and glucose application

The HEK293 cells were cultured in Dulbecco’s modified Eagle’s medium (DMEM, containing 5.5 mM D-glucose, 10% fetal bovine serum (FBS), 100 μg/ml streptomycin and 100 U/ml penicillin), and exposed to 50 mM of either D-glucose or L-glucose. After 48 h treatment, the cells were collected for immunoblotting.

### Western blot

The methods for protein extraction and immunoblotting used in this study were the same as those we described previously^48^,^49^. The HH10 Chick heads (N > 20 embryos in each group) and HEK293 cells were collected and lysed using the CytoBuster™ Protein Extraction Reagent (Novagen, #71009) containing proteinase and phosphotase inhibitors (Roche, cOmplete mini EDTA-free and PhosSTOP). The total protein concentrations were assessed using a BCA quantification kit (DingGuo BioTECH, BCA01). The samples containing identical amounts of protein were fractionated via SDS-PAGE and then transferred to PVDF membranes (Bio-Rad). The membranes were blocked with 5% Difco™ skim milk (BD, #232100) and subsequently incubated with primary and secondary antibodies; then, the bands representing the proteins of interest were visualized using an ECL kit (Thermo, #34079) and GeneGnome5 (SYNGENE). The intensity of the bands was analyzed using Quantity One software (Bio-Rad). The antibodies used for the western blots were as follows: P53 (Millipore, CBL404), Caspase7, cleaved-Caspase7, Caspase3, cleaved-Caspase3 (CST, #9915), LC3B, phospho-Akt-Ser473 (CST, #4060), phospho-ERK1/2 (CST, #4370), ERK1/2 (CST, #4695), Beclin1 (Santa Cruz, sc-11427), β-actin (Proteintech, #60008), Atg5 (Abgent, AP1812b), Akt (Abgent, AJ1022a), horseradish peroxidase (HRP)-conjugated anti-mouse IgG (CST, #7076), and HRP-conjugated anti-rabbit IgG(CST, #7074). All of the primary antibodies were diluted 1000-fold in 5% skim milk, and the secondary antibodies were diluted 3000-fold.

### Photography

Following immunostaining, the whole mount embryos and the regions of interest were photographed using a stereo-fluorescence microscope and processed using the Olympus software package Image-Pro Plus 7.0. The embryos were then sectioned into 10 μm thick slices using a cryostat microtome (Leica CM1900), photographed using an Olympus IX51epi-fluorescent microscope (at 200x and 400x) and analyzed using CW4000 FISH Olympus software.

### Data analysis

The data analyses and statistical charts were generated using Graphpad Prism 5 software (Graphpad Software, CA, USA). The results were presented as the mean value (Mean ± SEM). All the quantitative data were analyzed using one-way ANOVA to test the difference. And Chi square test was employed to analyze rate among the experimental groups. *P* < 0.05 was considered to be significantly different.

## Additional Information

**How to cite this article**: Wang, X.-Y. *et al.* High glucose environment inhibits cranial neural crest survival by activating excessive autophagy in the chick embryo. *Sci. Rep.* **5**, 18321; doi: 10.1038/srep18321 (2015).

## Supplementary Material

Supplementary Information
