# Supplementary material for: High glucose environment inhibits cranial neural crest survival by activating excessive autophagy in the chick embryo
Source: Sci Rep. 2015 Dec 16;5:18321. doi: 10.1038/srep18321 (PMC4680872; doi:10.1038/srep18321)
Supplement: Supplementary Information [file srep18321-s1.doc]

**High glucose environment inhibits cranial neural crest survival by activating excessive autophagy in the chick embryo**

*Xiao-Yu Wang^1,4^,* *Shuai* *Li^1^,* *Guang* *Wang^1^,* *Zheng-lai Ma^1^,* *Manli* *Chuai^2^,* *Liu Cao^3^, Xuesong Yang^1*^*

*^1^**Division of Histology & Embryology, Key Laboratory for Regenerative Medicine of the Ministry of Education,* *Medical College, Jinan University,* *Guangzhou 510632, China*

*^2^**Division of Cell and Developmental Biology, University of Dundee,* *Dundee, DD1 5EH, UK*

*^3^**Key Laboratory of Medical Cell Biology, China Medical University,* *Shengyang 110001, China*

*^4^Division of Pathophysiology, Medical College, Jinan University, Guangzhou 510632, China*

*^*^The corresponding author: Xuesong Yang (yang_xuesong@126.com)*


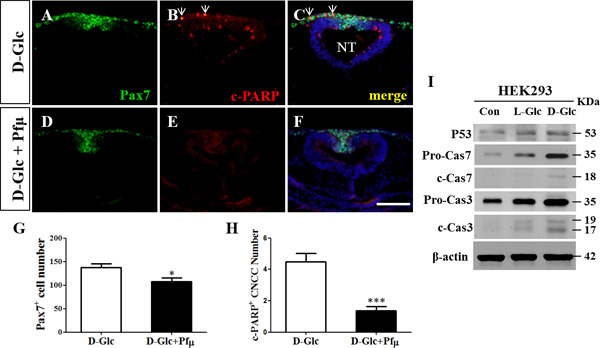


***Supplementary Figure-1: High glucose environment induced apoptosis in a P53-dependent manner in the cranial neural crest cells***

The chick embryos were exposed to D-glucose and D-glucose combined with Pfμ (20μM) until HH10 before being harvested for Pax7 and c-PARP immunofluorescent staining (A-F). **A-C**: The fluorescent images of the transverse section at the midbrain level show the expression of Pax7, c-PARP and merged one counterstained with DAPI. The apoptotic cranial neural crest cells were indicated by white arrows in B-C. N=10 embryos. **D-F**: In the D-glucose combined Pfμ treated group, few of apoptotic cranial neural crest cells were observed on the transverse sections. N=9 embryos. **G**: The bar chart showing the comparison of number of Pax7 positive cranial neural crest cells between the D-glucose and D-glucose combined with Pfμ treated embryos. **H**: The bar chart showing the comparison of number of c-PARP positive cranial neural crest cells between the D-glucose and D-glucose combined with Pfμ treated embryos. **I**: The human embryonic kidney 293 (HEK293) cell line was exposed to simple saline (control) and high glucose (L- and D-Glucose) for 48 hours. The expression of P53, Pro-Cas7, Cleaved-Cas7, Pro-Cas3 and Cleaved-Cas3 protein was determined by Western blot. **P*<0.05 and ****P*<0.001 indicate significant differences between the D-glucose and D-glucose combined with Pfμ treated embryos. Abbreviations: D-Glc, D-Glucose; Pfμ, Pifithrin-μ; c-PARP, cleaved PARP; NT, neural tube. Scale bars =100 µm in A-F.

Supplementary dataset 2


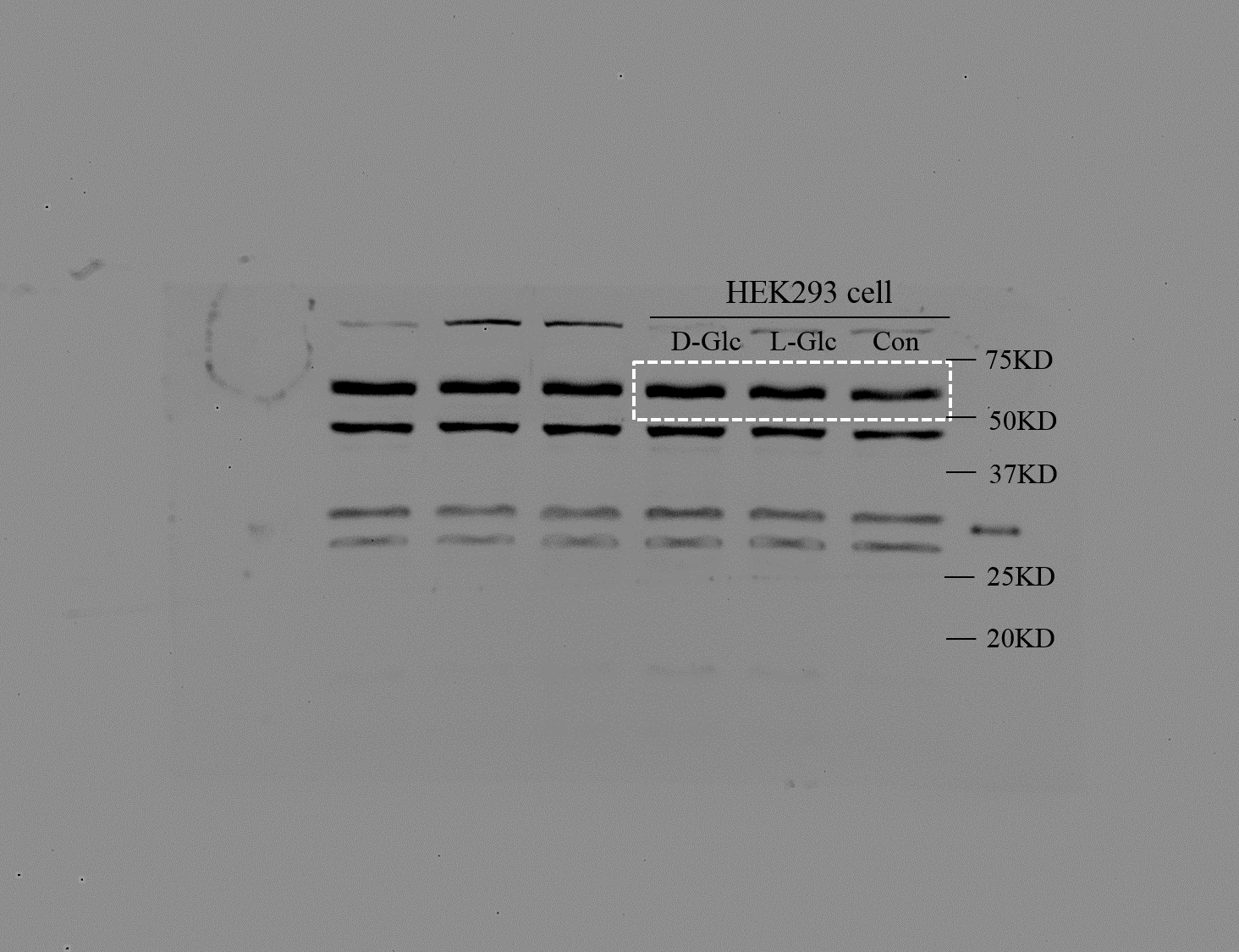


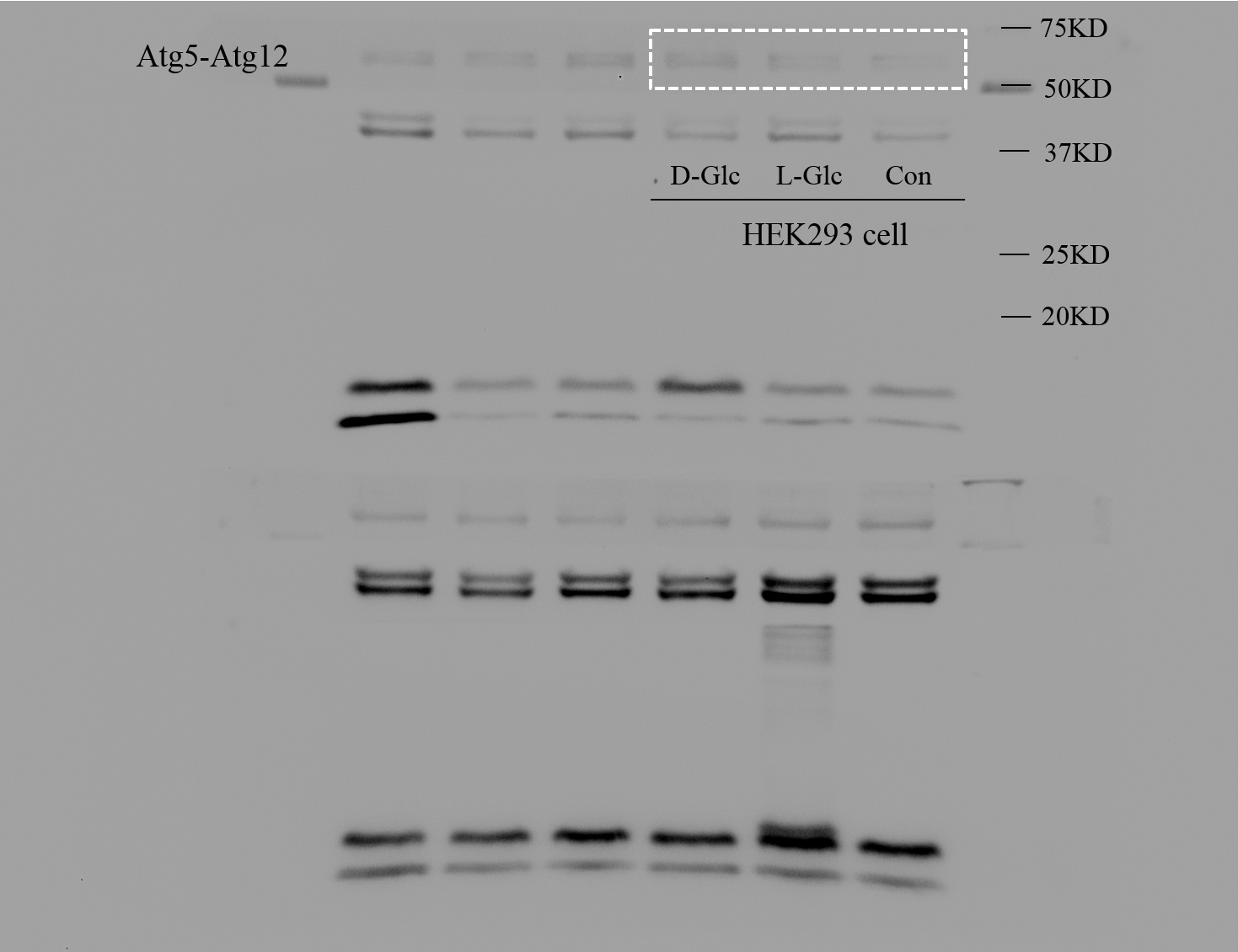


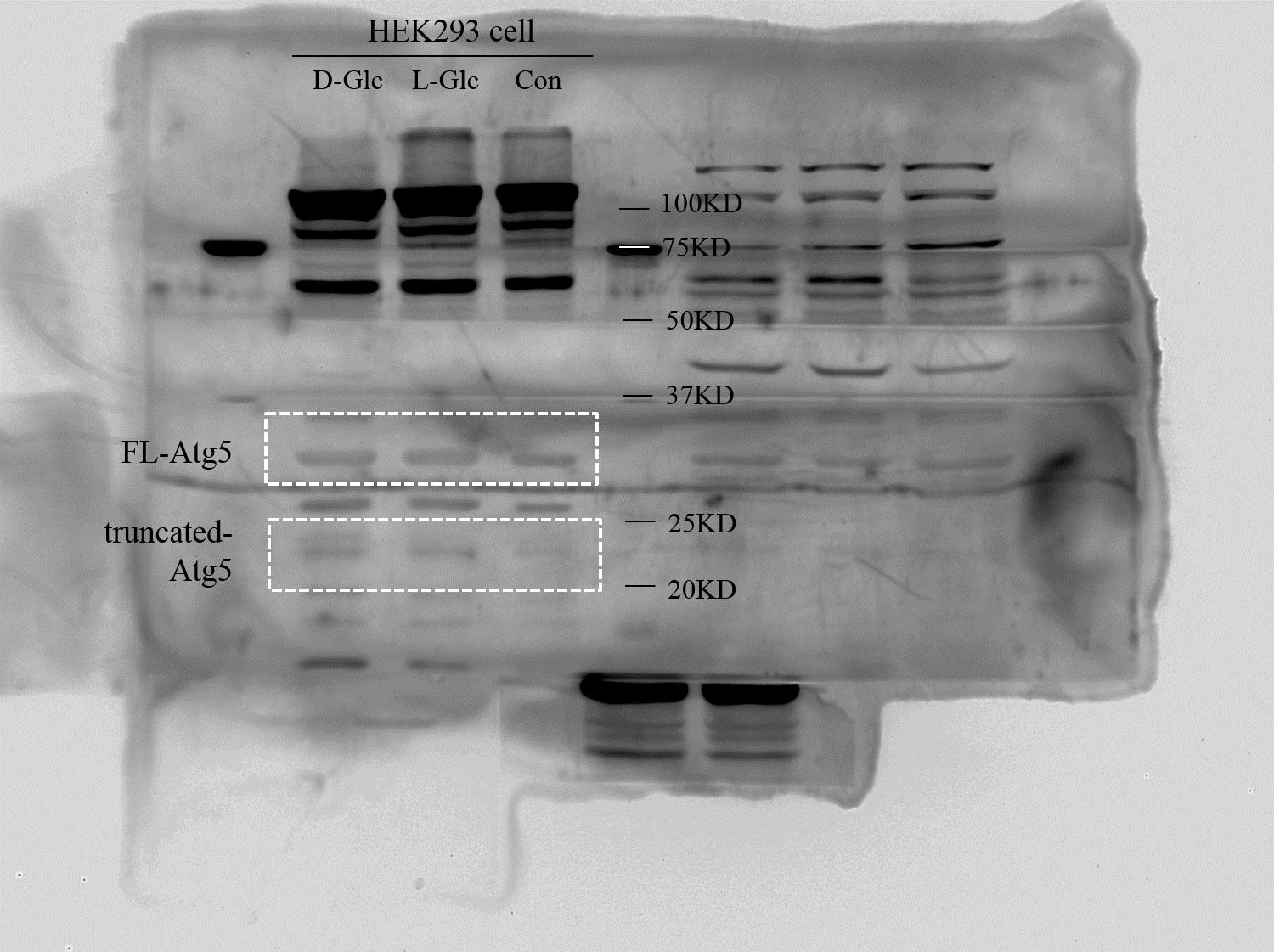


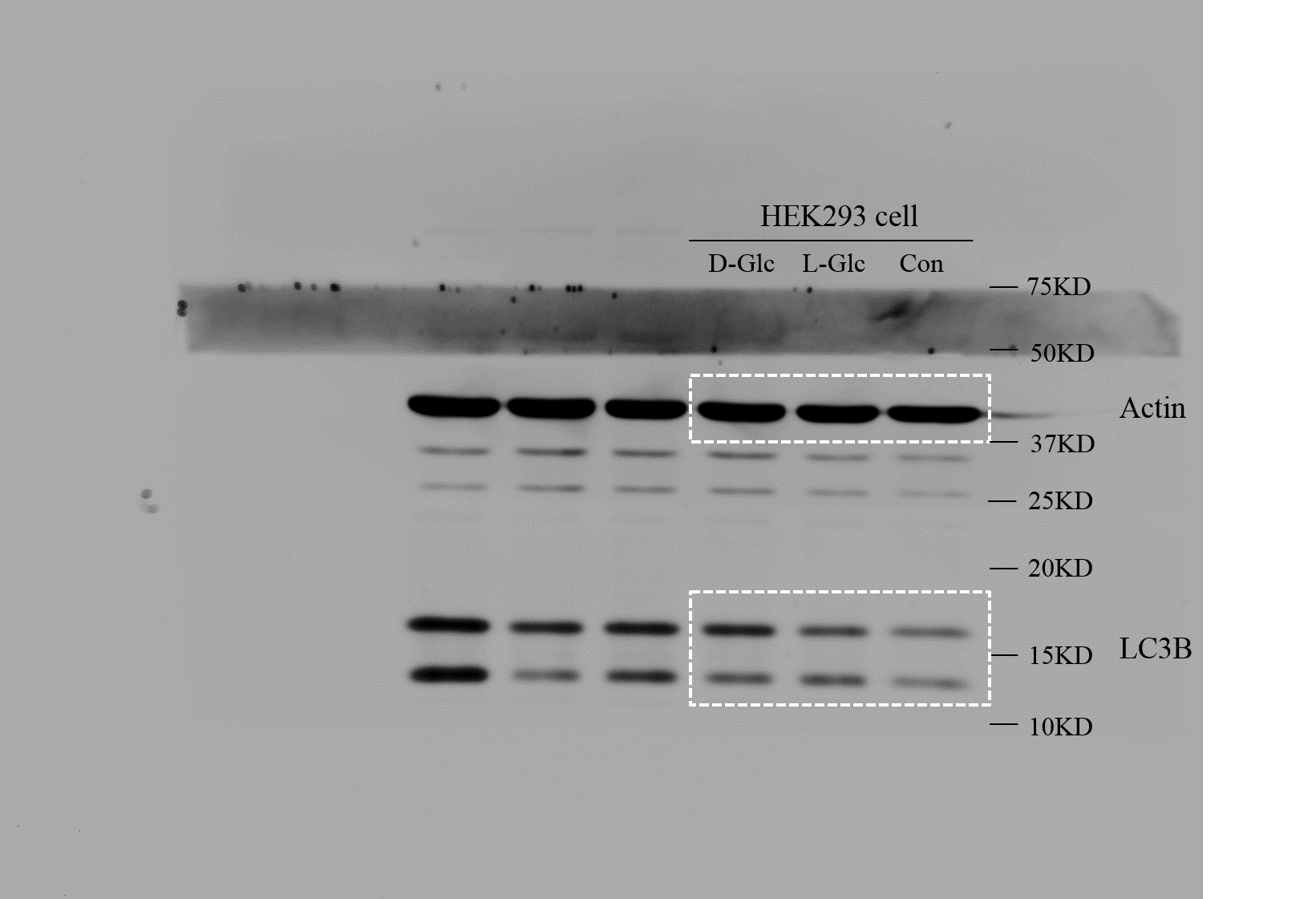


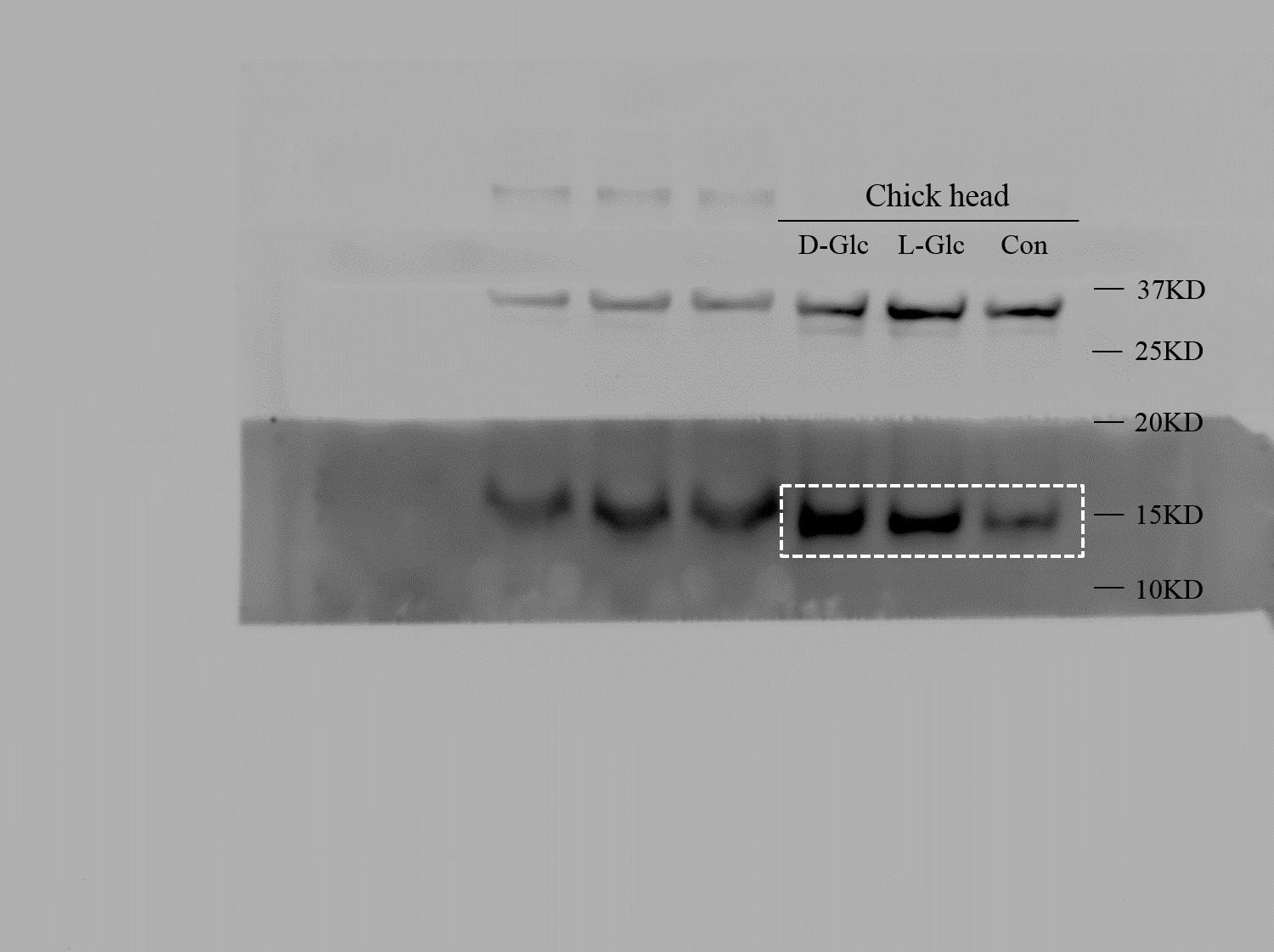


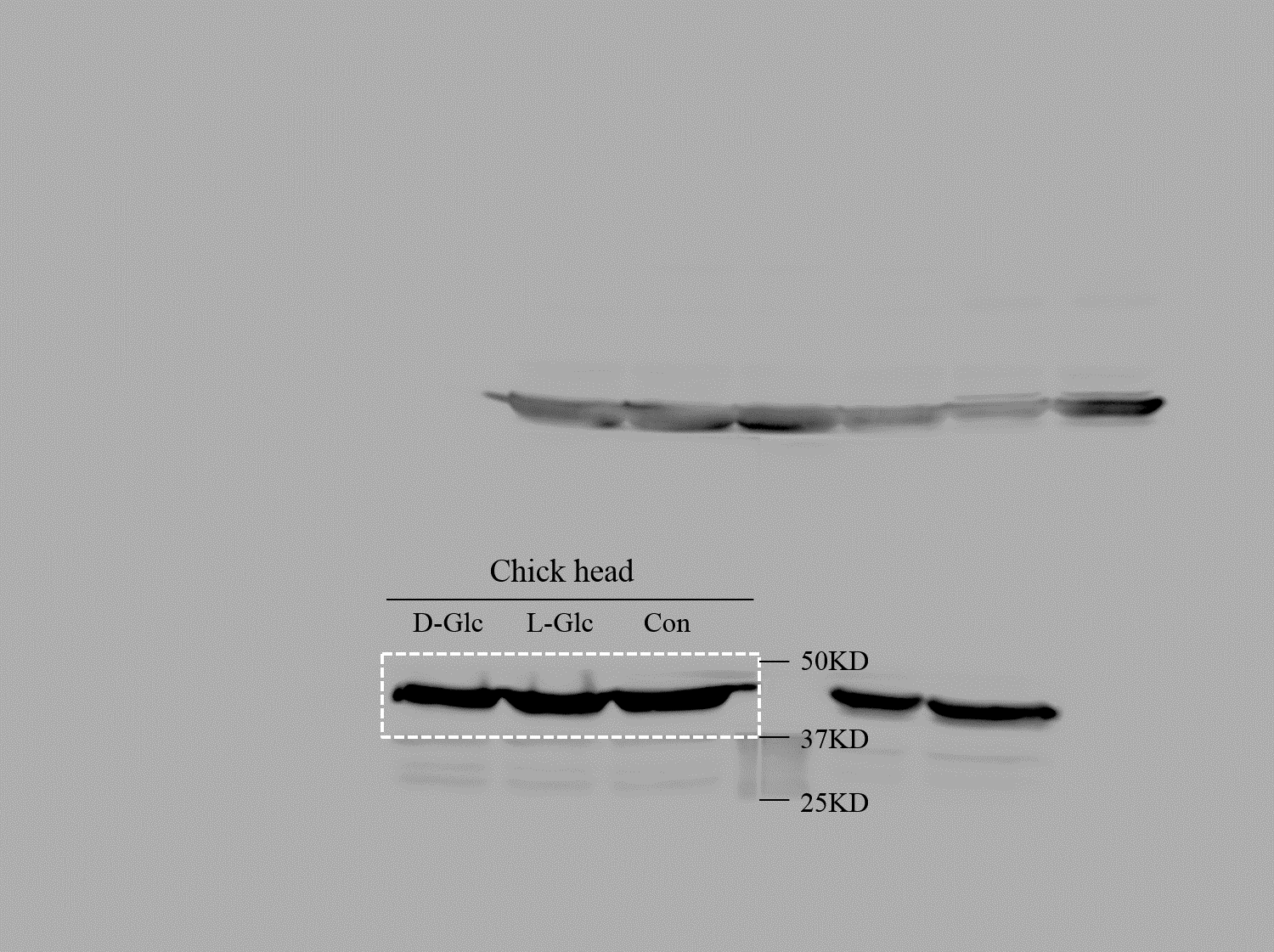


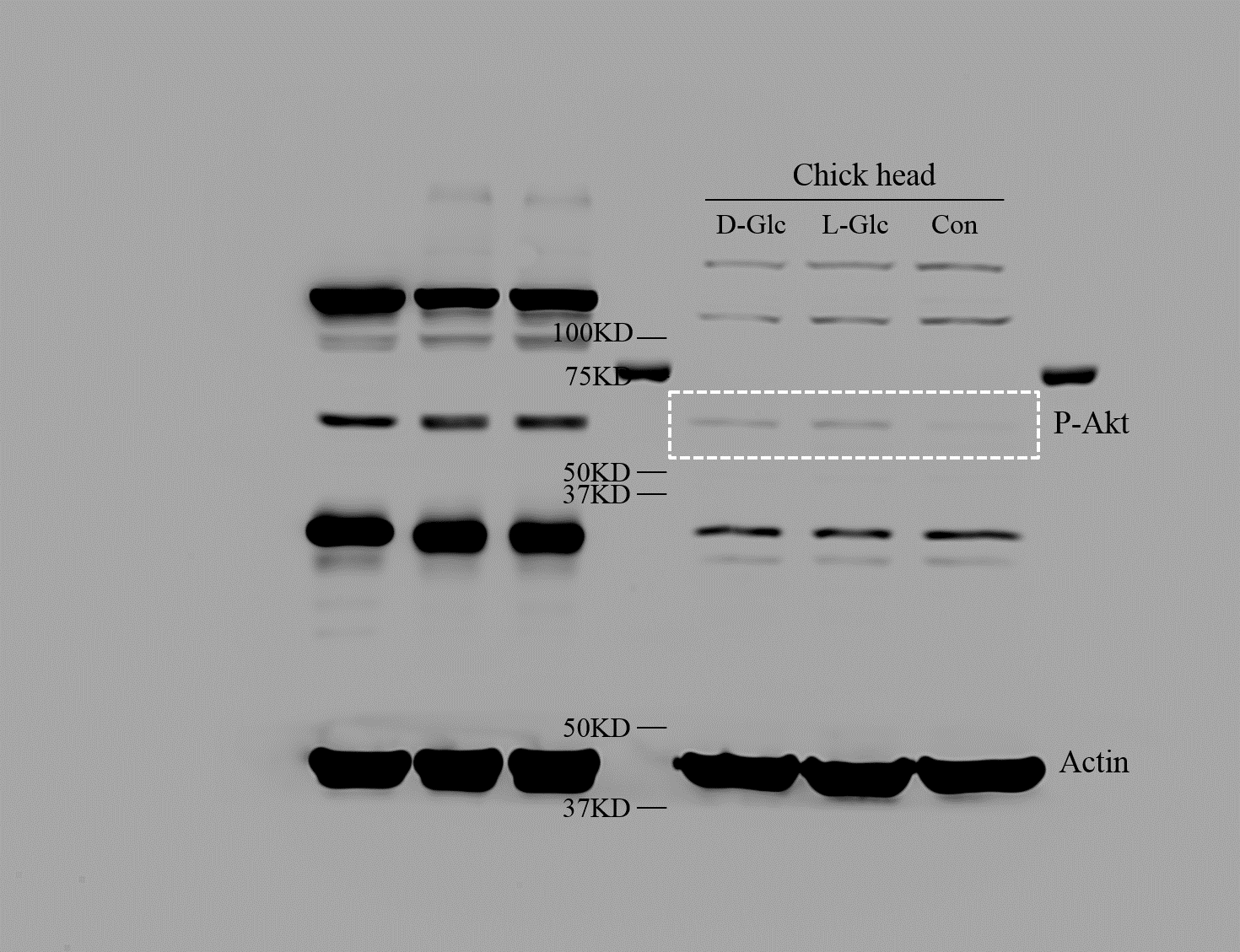


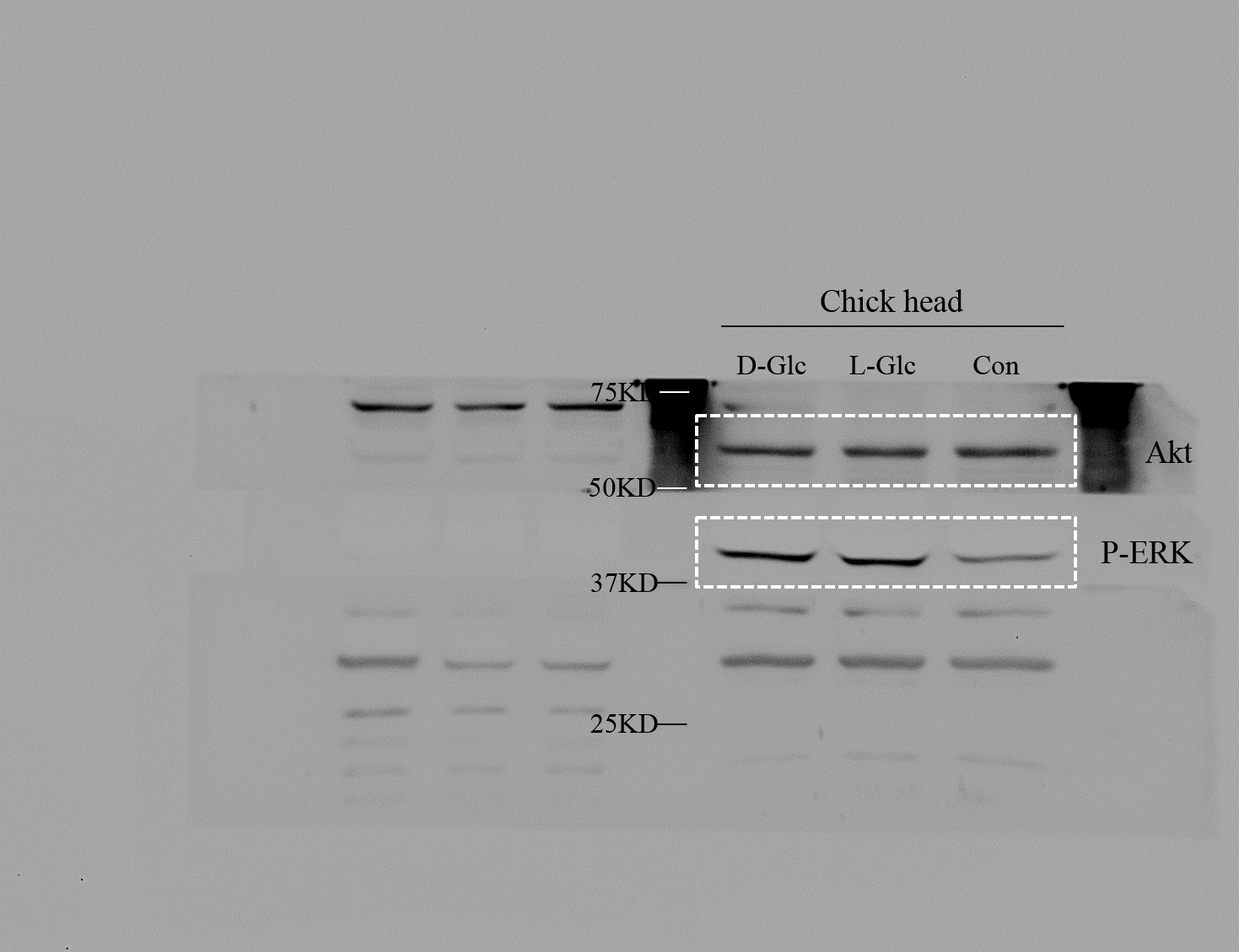


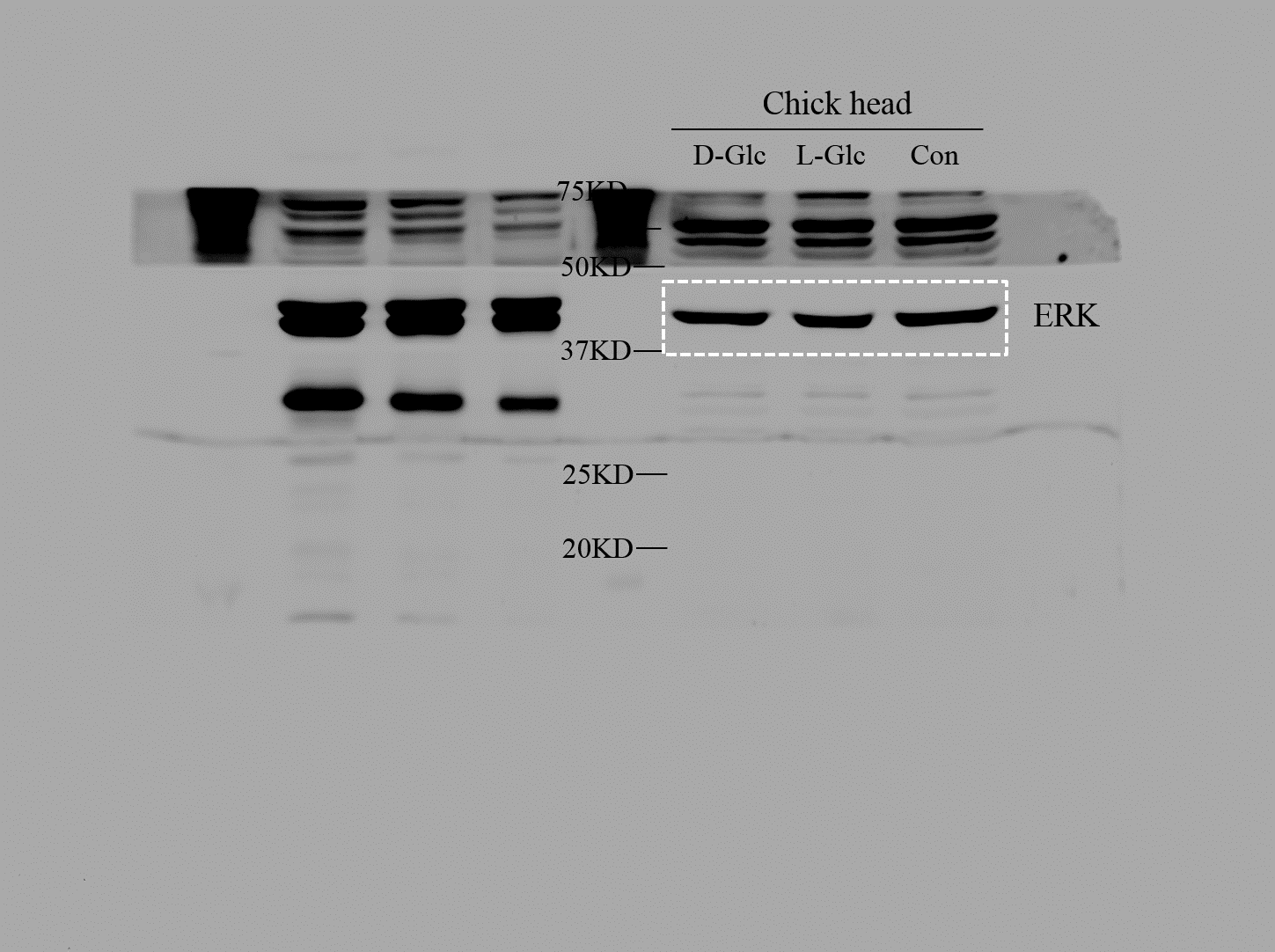


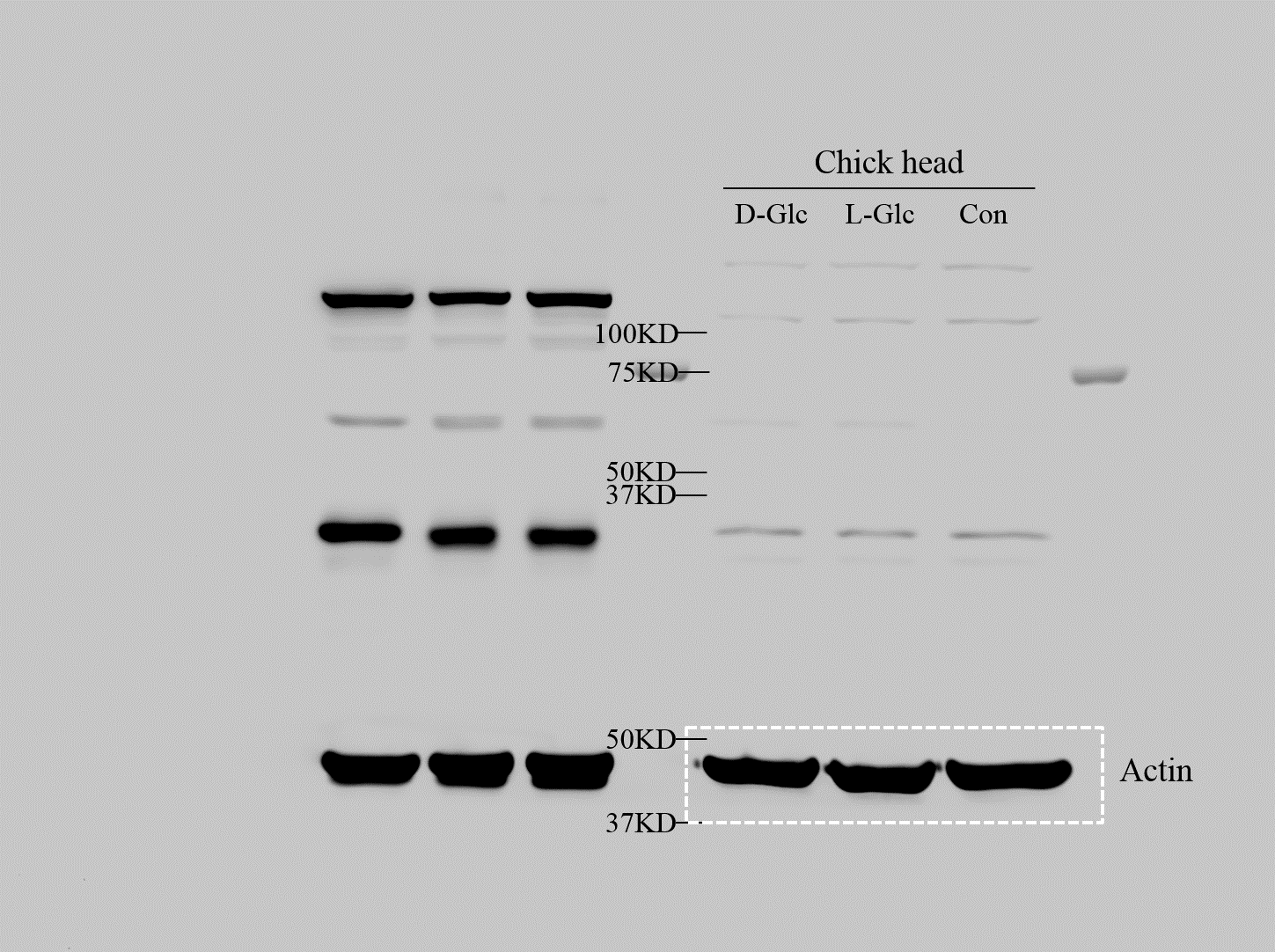


**
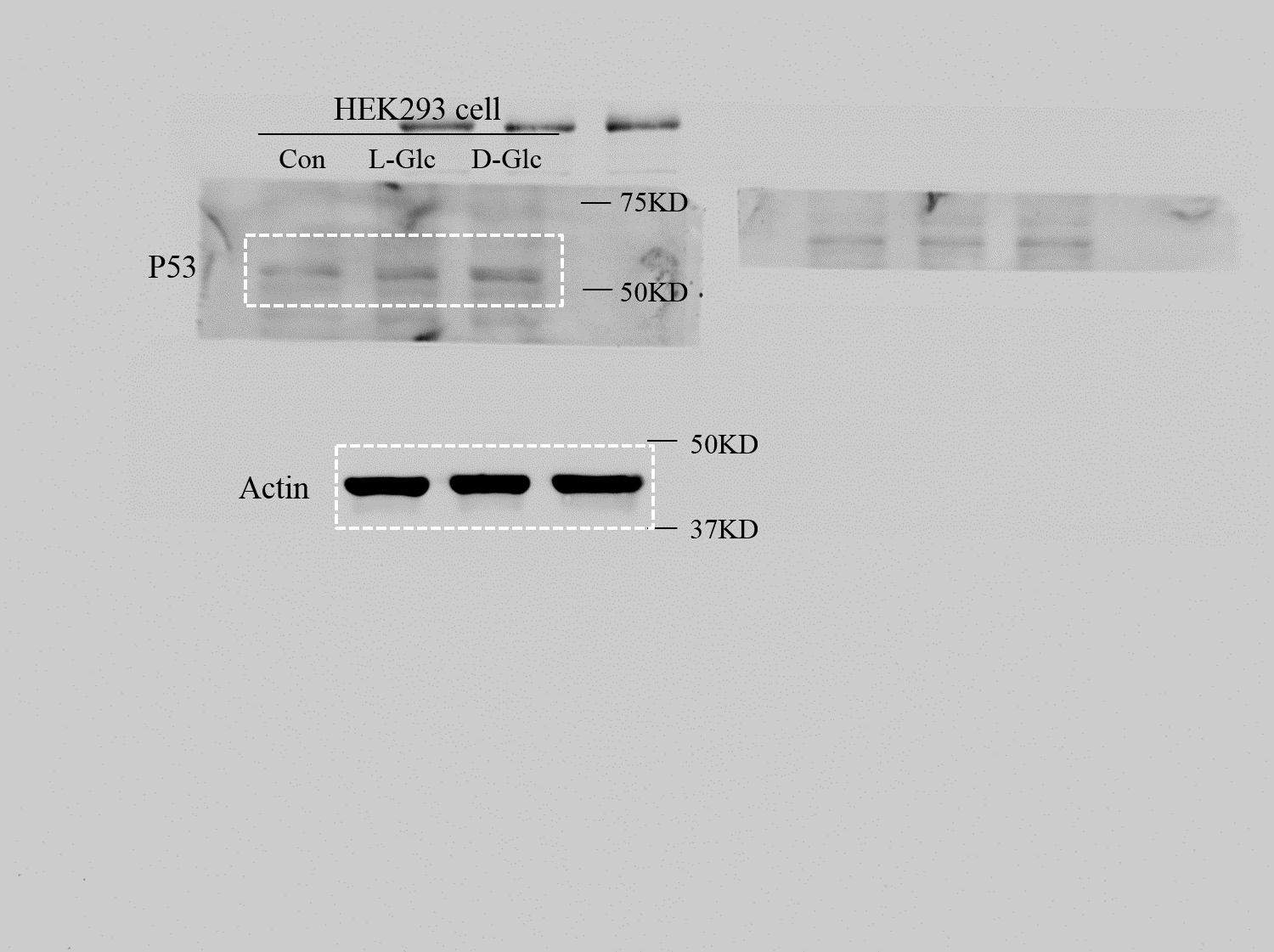
**

**
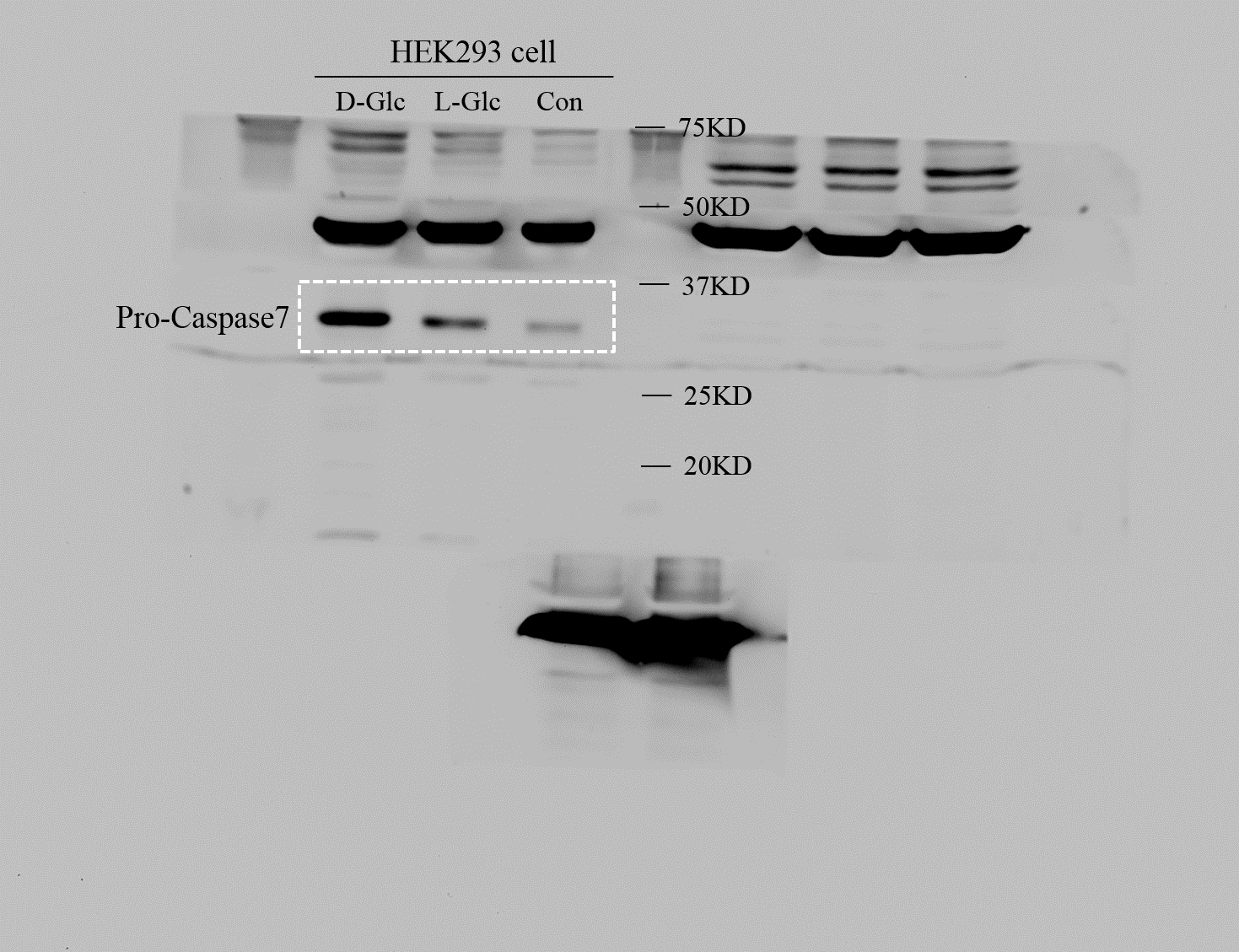
**

**
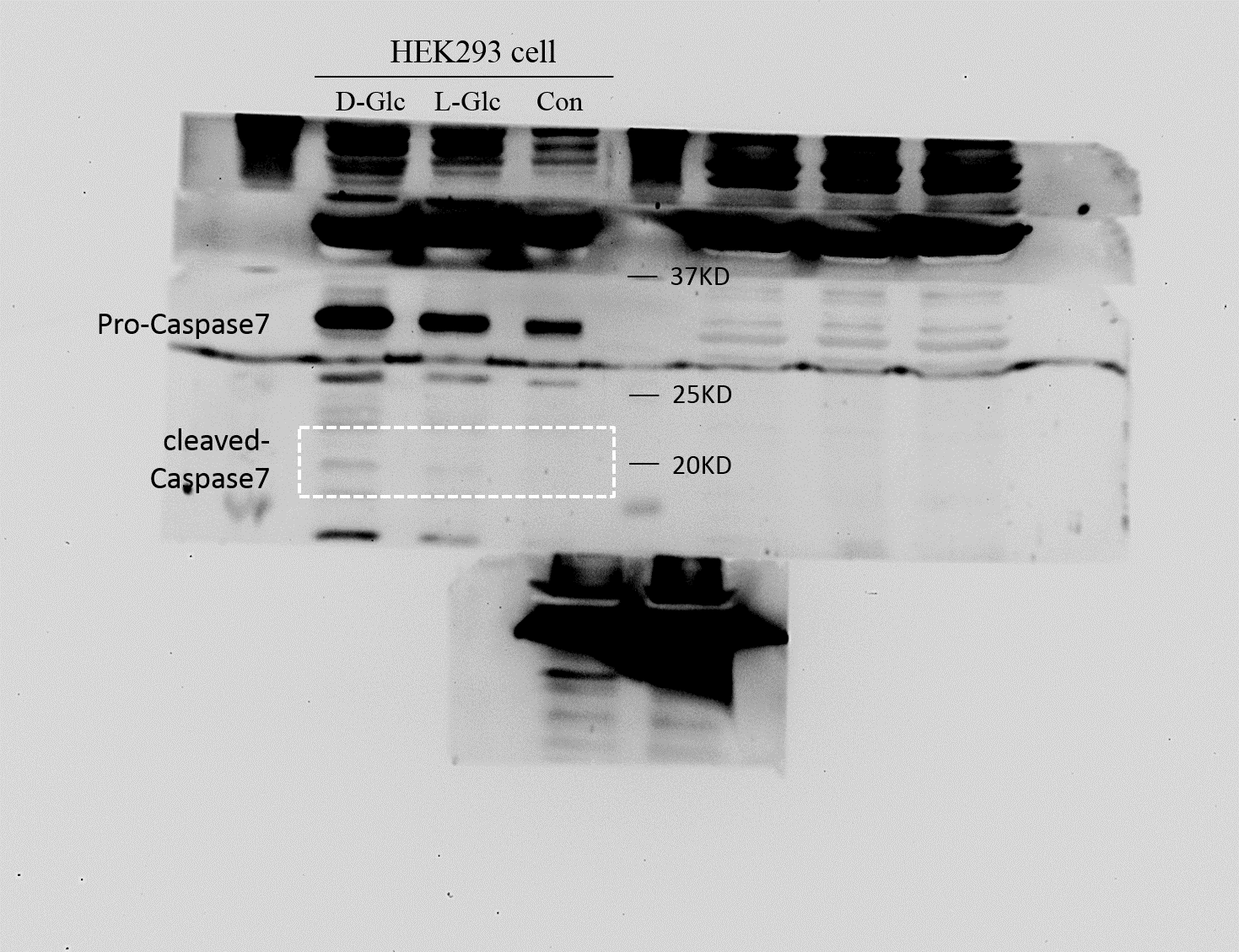
**

**
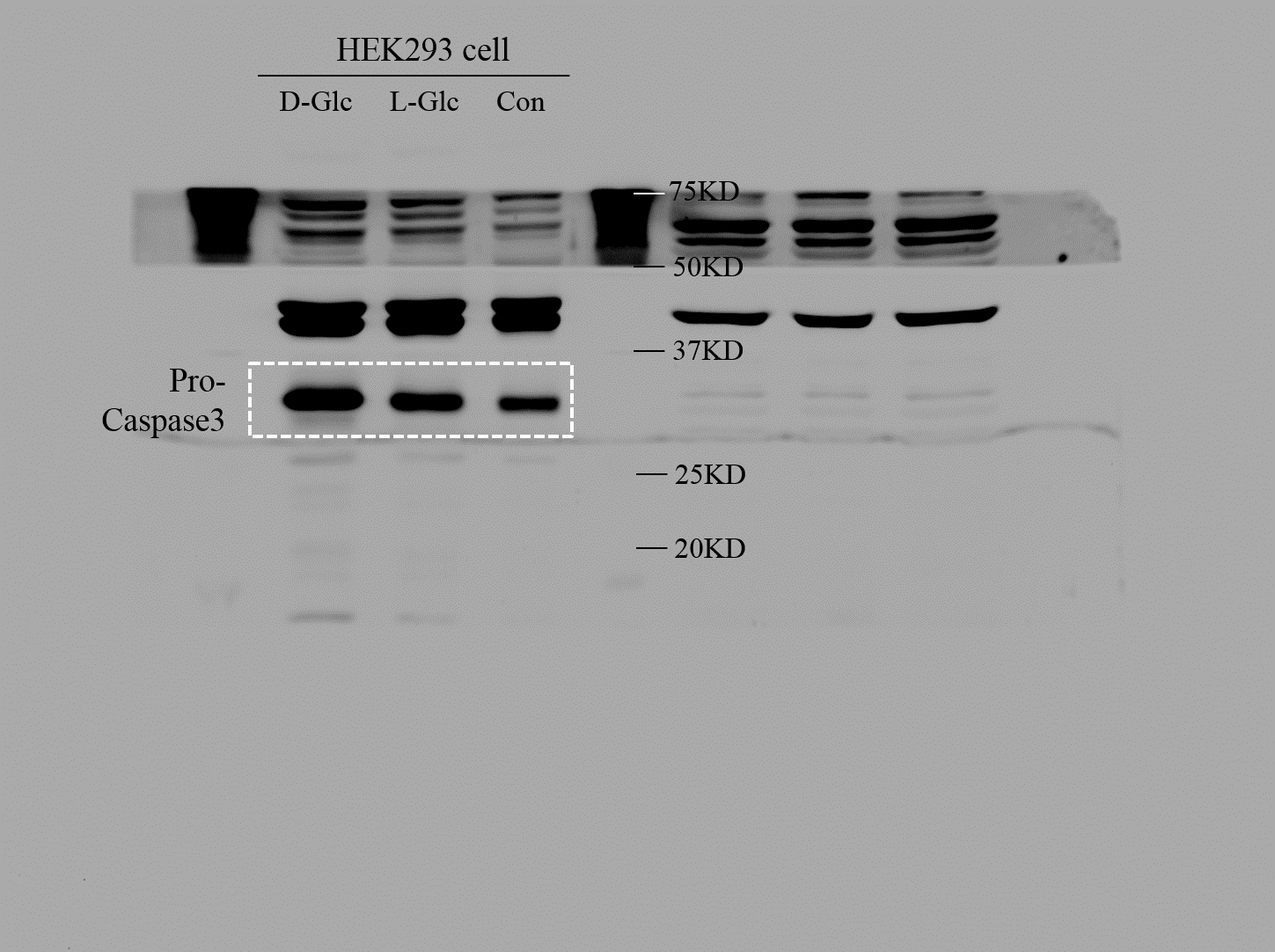
**

**
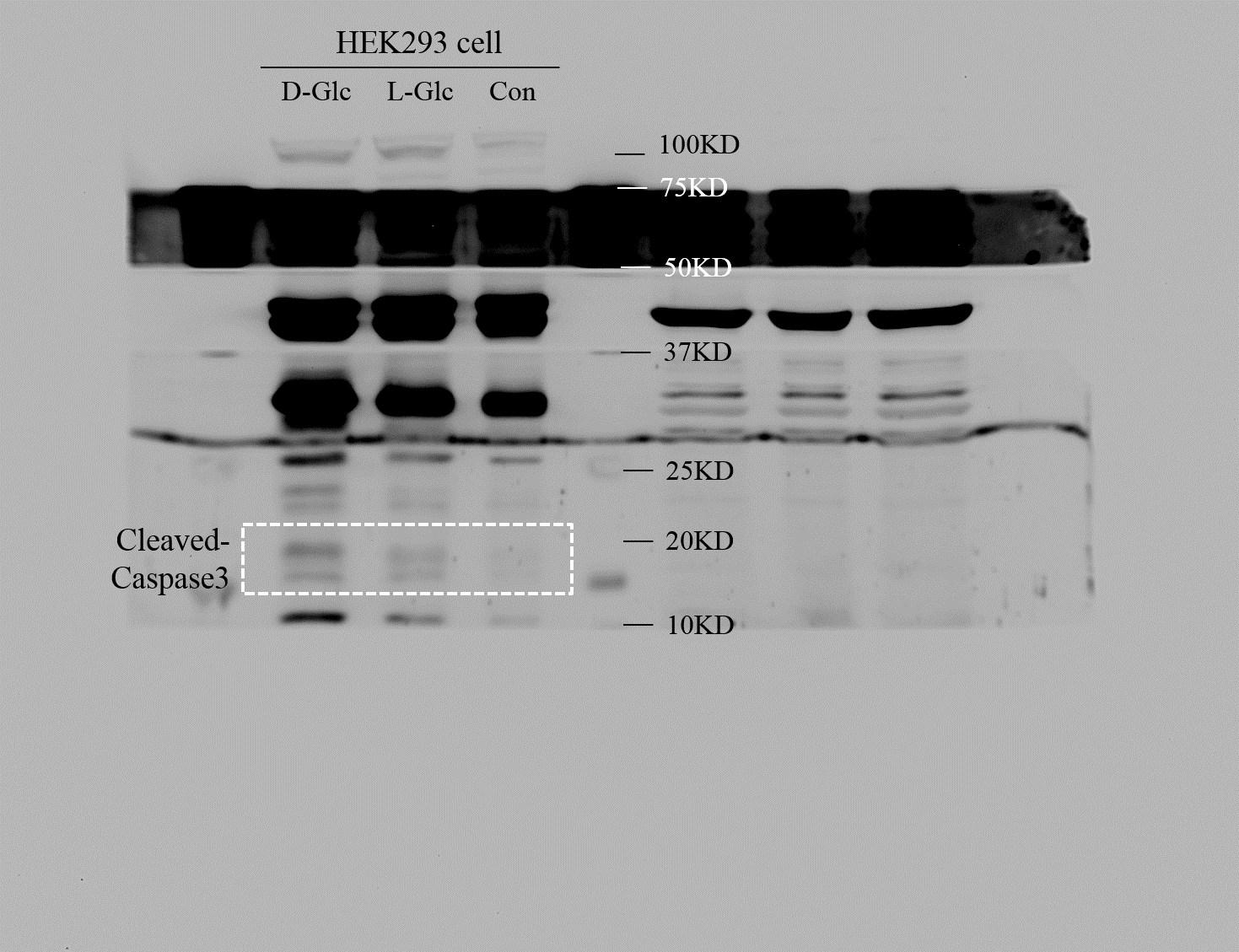
**
